# Spatio-temporal metabarcoding surveys in ports reveal homogenised communities of non-indigenous species with high genetic diversity and connectivity

**DOI:** 10.1038/s41598-026-49393-3

**Published:** 2026-04-26

**Authors:** J. Zarcero, A. Antich, M. Fernández-Tejedor, C. Palacín, O. S. Wangensteen, M. Rius, X. Turon

**Affiliations:** 1 Department of Marine Ecology, Centre for Advanced Studies of Blanes (CEAB), Spanish Scientific Research Council (CSIC), Blanes, Catalonia Spain; 2 Department of Evolutionary Biology, Ecology and Environmental Sciences and Biodiversity Research Institute (IRBio), University of Barcelona, Barcelona, Catalonia Spain; 3 Institute of Agrifood Research and Technology (IRTA), La Ràpita, Catalonia Spain; 4 Section of Biological Sciences, Institute of Catalan Studies (IEC), Barcelona, Catalonia Spain; 5 Department of Zoology, Centre for Ecological Genomics and Wildlife Conservation, University of Johannesburg, Auckland Park, Johannesburg, 2006 South Africa

**Keywords:** COI Metabarcoding, Intraspecific variability, Non-indigenous species, Metaphylogeography, Ports, NIS database, Ecology, Ecology, Evolution, Genetics, Ocean sciences, Zoology

## Abstract

Large commercial ports facilitate the introduction of non-indigenous species (NIS), while smaller harbours and marinas promote their regional spread, making harbour networks key drivers of biological invasions. We analysed spatio-temporal patterns of metazoan communities over one year in four medium-size harbours, as well as at an outside reference point, along the NW Mediterranean coast using standardised biological collectors and COI metabarcoding. We identified 1,774 metazoan molecular operational taxonomic units (MOTUs), of which 75 were classified as NIS. Although less diverse than native species, NIS accounted for 34-70% of reads in harbours, with the southernmost harbour showing the highest abundance, likely due to proximity to aquaculture facilities. Spatial structure varied among harbours, but NIS showed consistently low genetic differentiation and shared more MOTUs among sites than native species. Seasonal patterns affected both NIS and native communities. NIS also exhibited higher haplotype diversity and lower genetic differentiation across harbours, suggesting spread via local boating and recurrent introductions. These results highlight contrasting dynamics between NIS and native species in artificial environments and underscore the need for continued monitoring of harbour networks.

**Supplementary Information:**

The online version contains supplementary material available at 10.1038/s41598-026-49393-3.

## Introduction

Population connectivity is a key process shaping community structure^1,2^. Although most studies of population connectivity focus on natural habitats, increasing attention has been given to connectivity among highly impacted, anthropized ecosystems^3^. These studies often focus on a single or a small group of species^4^ and thus little is known about population connectivity among these ecosystems considering whole community composition.

Ports and marinas are emerging as ubiquitous elements of coastal seascapes, being part of the so-called coastal urban sprawl^5^. The continuous expansion of these artificial infrastructures profoundly impacts natural littoral communities and raises significant socio-ecological concerns^6^. Differences in biotic assemblages between natural and artificial habitats have been widely documented^7–10^. However, ports offer a unique setting for studying evolutionary responses to both novel population connectivity networks and changing environmental conditions^11,12^. Despite a recent focus on research in these ecosystems, there is a lack of studies addressing spatio-temporal patterns of community structure in port environments^5^.

Ports also serve as hotspots for the establishment and spread of non-indigenous species (NIS), which are a growing concern for a wide array of ecosystems^8,13–15^. Port communities are typically composed of species tolerant to changing biotic conditions, pollution, and other harbour-specific stressors, such as intense human activity or limited connections with the open system. Propagule pressure as a result of maritime traffic is a major driver of NIS introductions^16,17^, contributing to the richness of NIS in the Mediterranean Sea^18–20^. As such, ports are central to NIS dynamics and warrant focused studies^21–24^. While previous genetic studies on NIS in artificial environments have typically adopted a species-by-species approach^25–30^, community-level comparisons that include both native and NIS have the potential to shed light on the metacommunity-level population connectivity of harbour ecosystems.

Although taxonomy-based field surveys greatly aid NIS detection in ports^31–33^, species identifications can be time-consuming, often require specialised expertise, and can be particularly complex in taxonomically challenging groups such as ascidians, bryozoans, and cnidarians^34–37^. Consequently, molecular methods, especially metabarcoding, are increasingly used for port community monitoring^38–40^. These tools generate high-throughput biodiversity data that can reveal whole-community composition, detect NIS, and monitor temporal change^41^. Nonetheless, challenges remain for metabarcoding studies, such as methods’ standardisation and the choice of markers and sampling substrates^38,40,42^. Thus, further development of reference databases and bioinformatic pipelines are crucial for enhancing the reliability of metabarcoding in NIS-focused harbour studies^43^. Adequate geographic and temporal coverage is also critical for assessing connectivity and seasonal trends^40,41,44^.

Genetic and genomic studies at intraspecific level, typically targeting one or a few NIS^26,45–47^, provide insights into invasion processes such as the so-called genetic paradox: invasive species often succeed despite reduced genetic diversity resulting from bottlenecks during introduction^48,49^. Multiple introductions may counteract this, enhancing genetic richness and promoting anthropogenic homogenisation^46,50^. Understanding NIS genetic diversity can therefore shed light on their invasion dynamics and the impacts on native species^51^. Metabarcoding data also allow for metaphylogeographic analyses that allow extracting intraspecific genetic signals such as haplotype composition^52^, and computing genetic differentiation measures among populations from denoised sequence variants^53–55^. This enables comparisons of genetic richness and connectivity between native and NIS’ populations, helping to explain differences in fitness, adaptability, and dispersal success^56,57^.

In this study, we analysed metazoan communities across four similarly sized ports characterised by fishing and leisure activities. We specifically avoided large commercial ports to concentrate on post-border processes - i.e., secondary spread via local boating and fishing^58–61^. By examining community structure and comparing native versus NIS dynamics, we explored both interspecific and intraspecific patterns, detailed in^52^, with special attention to unravelling patterns of genetic diversity and population connectivity. Furthermore, we included a natural site outside one of the harbours that provide a reference point to assist in the interpretation of the observed patterns. Our general expectations are that ports act as networks facilitating the connectivity of NIS^21,62^ and that recreational boating between ports leads to recurrent introduction of NIS. We specifically tested the following working hypotheses: 1) NIS communities will have higher homogeneity (i.e., lower beta-diversity) between ports than the overall community; 2) NIS will display greater levels of genetic diversity than native species and 3) NIS will exhibit higher population connectivity and less genetic differentiation than native species. Our approach will allow assessing how dispersal dynamics and propagule pressure influence the invasion processes and the resilience of native species.

## Results

### Global diversity and datasets

After pairing, demultiplexing, quality and length filtering, and chimera removal, we obtained 283,820,787 reads from 3,013,217 unique COI sequences. The denoising procedure resulted in 41,792 ESVs, which were grouped into 9,006 MOTUs. After the filtering steps adopted, 4,260 MOTUs and 24,782 ESVs were kept. From these, we retained all those assigned to marine metazoans. Ten samples were left with less than 9,500 reads and were discarded. Our final database thus consisted of 110 samples (Table S1) with a total of 1,774 MOTUs, 11,193 ESVs, and 123,883,837 reads. The final MOTU table, with taxonomic identification and assignment to the different datasets, is presented in Table S2. The final ESV table, with indication of the MOTU to which each ESV belongs, is given as Table S3. The rarefaction curves (Fig. S1A) showed that a plateau was reached in the number of MOTUs in each sample, indicating adequate sequencing depth. Conversely, the MOTU accumulation curves (Fig. S1B), which represent the cumulative number of MOTUs detected as additional samples are included, did not reach a plateau, with new MOTUs continuing to be added as more samples were analysed.

We detected 75 MOTUs belonging to NIS using BLAST with the NISdb, comprising 68,713,153 reads. These reads were distributed across 52 nominal species (Table S2). When we checked the taxonomy obtained with NISdb with the one obtained from the COInr database with mkLTG, they were coincident in 73 MOTUs, while for 7 MOTUs the mkLTG procedure could assign them only at genus or higher levels, for 2 MOTUs the mkLTG assignment was to a different species, and for another three it was to the same species but with less than 97% identity. The COMM dataset comprised the remaining 1,699 MOTUs and 55,170,684 reads. Of these, 244 MOTUs assigned at the species level were identified as native, with 24,323,826 reads and belonging to 218 nominal species (Table S2). All analyses have been performed at the MOTU level (not nominal species), unless otherwise stated.

### Taxonomic composition

**Fig. 1:**
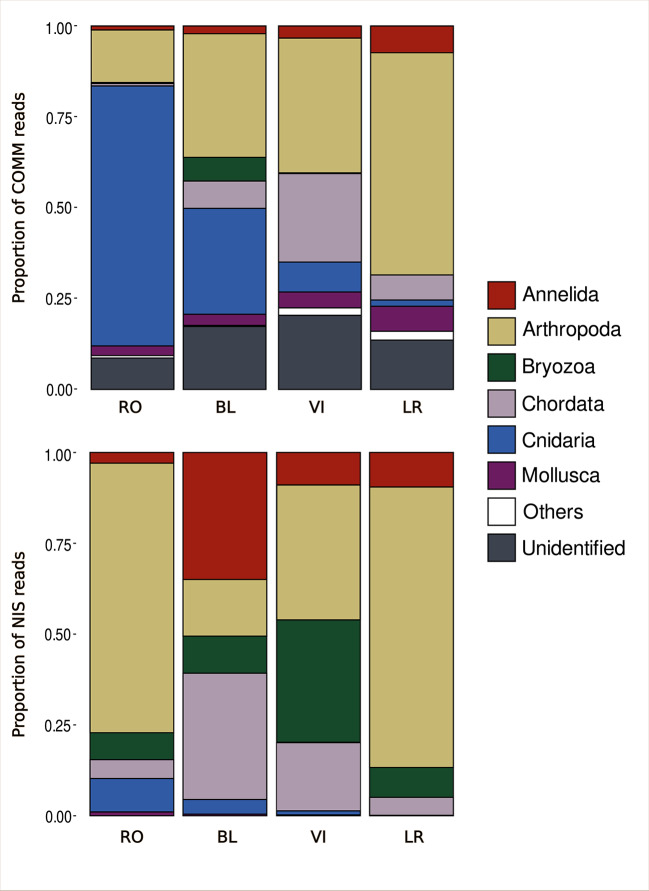
Barplots of the relative proportion of COMM (**A**) and NIS (**B**) reads of the different phyla for each area (RO: Roses; BL: Blanes; VI: Vilanova i La Geltrú; LR: La Ràpita).

Bar charts were prepared to show the overall taxonomic composition separately for each locality in terms of metazoan phyla composition for the COMM and NIS datasets (Fig. 1). The proportions were calculated based on the averaged relative abundances of the reads per locality. Phyla representing less than 5% of the reads were grouped under "Others”. The “Unidentified” category corresponded to metazoan reads that could not be assigned to phylum level. The group with the highest relative abundance in the COMM dataset were the arthropods, with cnidarians second. These phyla showed contrasting relative abundance trends, increasing from North to South for the former and decreasing for the latter. The third most abundant group were chordates (mostly ascidians), followed by annelids, molluscs, bryozoans, and “Others”. The “Unidentified” category had between 2% and 60% of the reads across all samples. When considering the NIS dataset, arthropods, annelids, cnidarians and chordates were, in this order, the dominant phyla in terms of relative read abundance, with marked differences across ports. For instance, BL had a higher abundance of annelids and chordates, while arthropods were the dominant group in the other ports, particularly RO and LR (>70% of NIS reads).

The taxonomic composition of the samples is presented separately for each sampling time for the COMM and NIS datasets in Figs. S2 and Figs. S3 respectively. For the COMM dataset, in northern ports (RO and BL), there was a trend of dominance of arthropods in spring-beginning summer, with higher relative abundance of cnidarians in late summer to winter. In turn, southern ports (VI and LR) showed that arthropods were in general the best represented group all year round (with the exception of July 2019), and cnidarians were not abundant, except in May 2019 in VI. The time course of relative abundances in the NIS dataset did not show a common pattern, with arthropods dominating most of the year in the northernmost (RO) and southernmost (LR) sites, and a strong presence of annelids in summer-fall in BL, and of bryozoans in fall-winter in VI. Chordates (mostly ascidians) were particularly abundant in BL and VI, but did not show a clear temporal pattern.

### ɑ-diversity

**Fig. 2:**
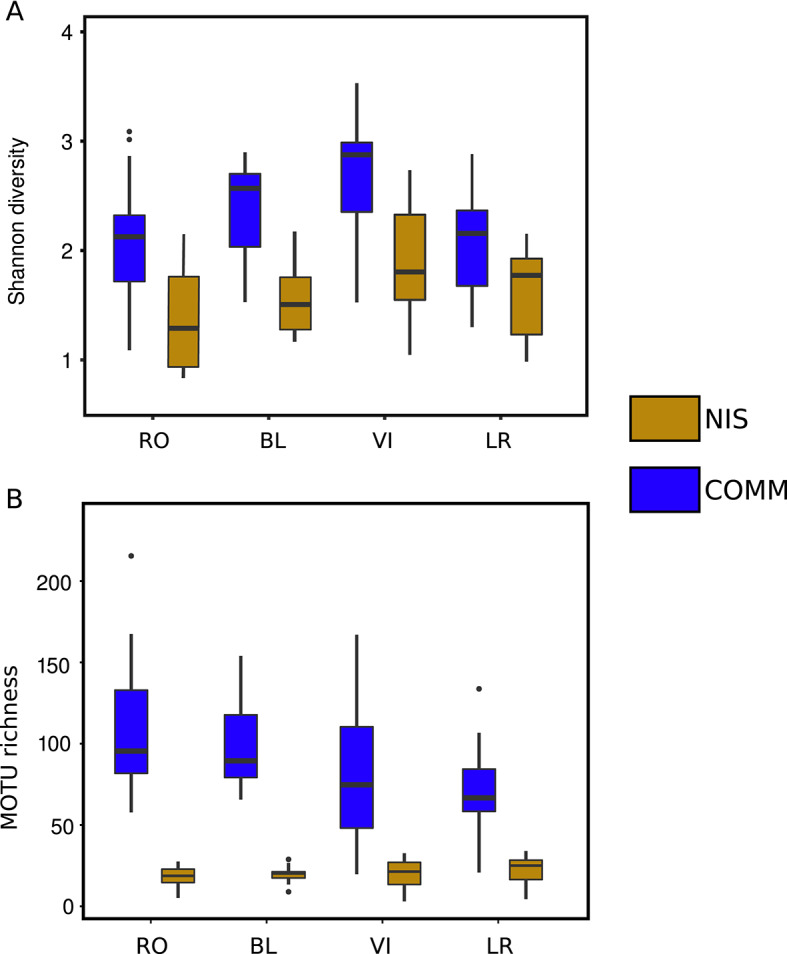
Box-plots of the values of Shannon diversity (**A**) and MOTU richness (**B**) of the COMM and NIS datasets. Horizontal lines are medians, boxes encompass the first and third quartiles, whiskers indicate 10th and 90th percentiles, and outliers are indicated as dot symbols. Site abbreviations are: RO - Roses, BL - Blanes, VI - Vilanova i La Geltrú, LR - La Ràpita.

The mean richness and Shannon diversity values after rarefaction (at 9,751 reads, the minimum number of reads of any sample) are shown in Fig. 2. For richness, RO had the highest values in the COMM dataset and the lowest values in the NIS dataset. For the COMM dataset, the mean diversity values were 2.33 ± 0.56 (mean ± SD) overall and the richness mean was 73.45 ± 32.18 MOTUs. For the NIS dataset, the mean diversity value for the rarefied data was 1.38 ± 0.48, and the mean richness value was 15.98 ± 4.21.

The analyses of variance showed a significant effect of locality on Shannon diversity in both datasets (Table S4A). Post-hoc (Tukey) tests revealed significant differences in the COMM dataset for the comparisons VI-RO, and LR-VI. For the NIS dataset, the lowest diversity values across ports were found in the northernmost port (RO), while VI had the highest diversity. No comparison between ports was significant except VI with RO. For MOTU richness, RO had the highest values across ports in the COMM dataset, and the southern ports of VI and LR the lowest. Locality was significant for the COMM dataset, and the post-hoc (Dunn) tests revealed significant differences in all comparisons between the northern (RO, BL) and the southern (VI, LR) communities. For the NIS dataset, the locality factor was not significant.

### Distribution patterns and β-diversity

**Fig. 3:**
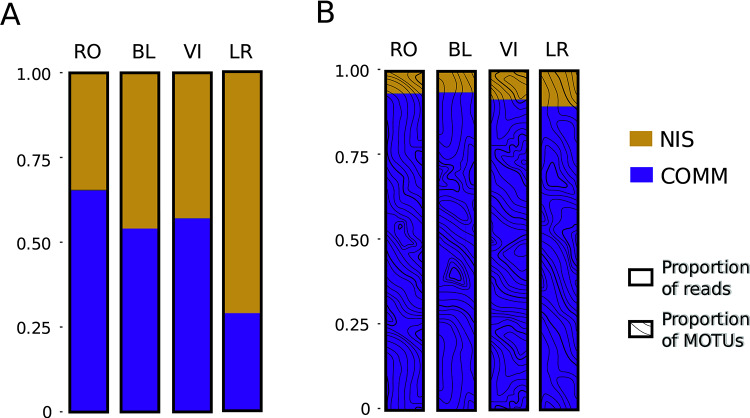
Barplots of the relative proportion of reads (**A**) and operational taxonomic units (MOTUs) (**B**) for the COMM and NIS datasets.

**Fig. 4:**
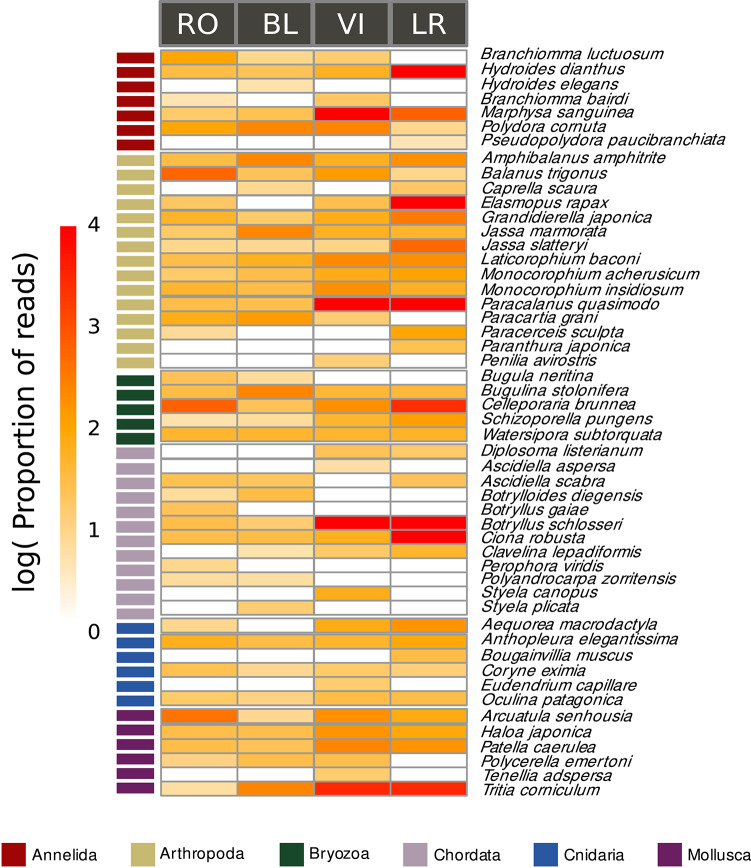
Heatmap showing relative read abundances (log-transformed) of the identified NIS in each area. Note that we have pooled molecular operational taxonomic units (MOTUs) assigned to the same nominal species. The phyla of the NIS are coded by colour. Blank cells indicate the absence of the species.

The relative read abundances of NIS were lowest in the northernmost port (RO) and highest in the southernmost port (LR), with intermediate values in BL and VI (Fig. 3). NIS always constituted more than 34% of the relative read abundance in each port, reaching ca. 70% in LR (Fig. 3A). However, in terms of the number of MOTUs (instead of reads), the proportion of NIS was minor compared to the total MOTUs of the entire community in all cases (Fig. 3B). In the locality with the highest proportion of NIS MOTUs (LR), they reached only 10% of the total. There was again an increasing trend from north to south, with the lowest proportion of NIS MOTUs in the northern ports, RO and BL (ca. 6%). Fig. 4 depicts the abundance of each NIS (MOTUs belonging to the same nominal species pooled) across localities, again reflecting their higher abundance in the southern ports.

**Fig. 5:**
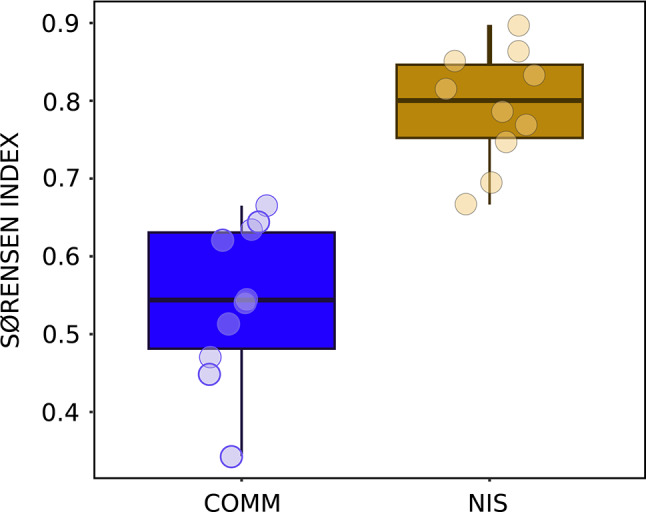
Box-plots of Sørensen index values for COMM and NIS MOTUs. Horizontal lines are medians, boxes encompass the first and third quartiles, whiskers indicate 10th and 90th percentiles.

Upset plots with the number of shared MOTUs (Fig. S4), showed markedly different patterns between COMM and NIS datasets. The majority of MOTUs of the former (66.7%) were unique to each locality (Fig. S4A). In contrast, the majority of NIS MOTUs (62.2%) were shared between at least two localities, and ca. one third were present in all four zones (Fig. S4B). The abundance of shared MOTUs between the different pairs of localities was also assessed with the Sørensen index (Fig. 5). The NIS MOTUs had a mean index of 0.828 among locality pairs, while the COMM MOTUs had a lower value (0.537), indicating less MOTU sharing, and the difference was significant (paired-sample *t*-test, p<0.001).

**Fig. 6:**
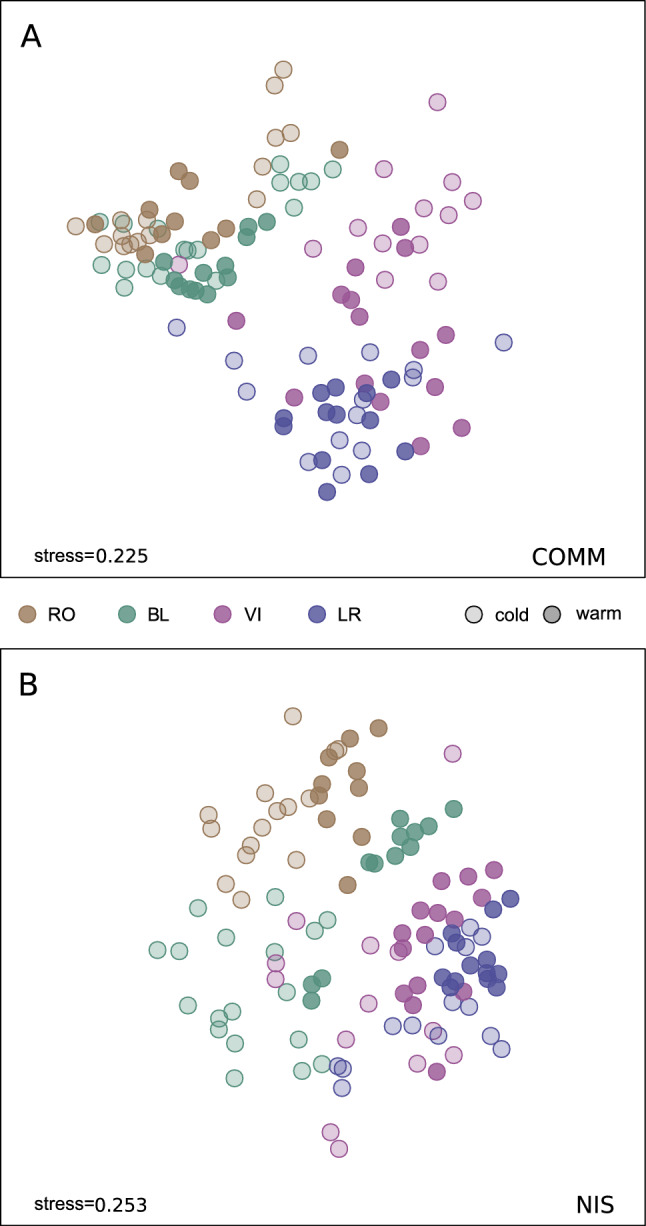
NMDS configurations for COMM (**A**) and NIS (**B**) datasets. Localities are indicated in different colours, and shading differentiates cold from warm months (applying a threshold of 20ºC, solid colours corresponding to warm periods). Stresses of the final configurations are indicated.

Regarding the nMDS for the COMM datasets (Fig. 6A), the ordination along the first axis separated the four ports into two groups, the northern ports (RO and BL) and the southern ones (VI and LR). For the NIS dataset (Fig. 6B), the overall pattern was similar, but featuring less distinct clusters and more overlap. The mean BC dissimilarity values between pairs of samples was significantly higher for COMM than for NIS (mean of 0.776 and 0.688, respectively, paired-sample *t-*test, p<0.001).

The PERMANOVA tests indicated significant effects of site and month for both COMM and NIS MOTUs (Table S4B). The permdisp tests revealed significant differences in multivariate dispersion for the site factor in both datasets, but not for the month factor. Pairwise tests showed significant differences in both cases for all pairwise comparisons between sites. For the month factor, the COMM dataset had more significant pairwise comparisons than the NIS dataset. July was the month most differentiated from all others in both datasets. Consecutive sampling times were not significantly differentiated, except for June-July in both datasets and July-August in the NIS dataset.

Temperature of the sampled localities (Fig. S5) showed clear-cut seasonal patterns, with lower summer temperatures in the northern ports (RO, BL), but lower winter temperatures in the South (LR, VI). Accordingly, some separation of cold and warm months, albeit with overlap, is also apparent in the MDS configurations for each site, both for COMM and NIS datasets (Fig. 6). We also plotted the mean Bray-Curtis dissimilarities values between consecutive sampling times separately for the COMM and NIS datasets (Fig. S6). For NIS these values peaked from July to February, indicating a higher species turnover, and were lowest in spring-beginning summer, suggesting more stability. The COMM dataset had overall higher BC distances and a similar temporal pattern.

### Intra-MOTU genetic diversity and metaphylogeography

**Fig. 7:**
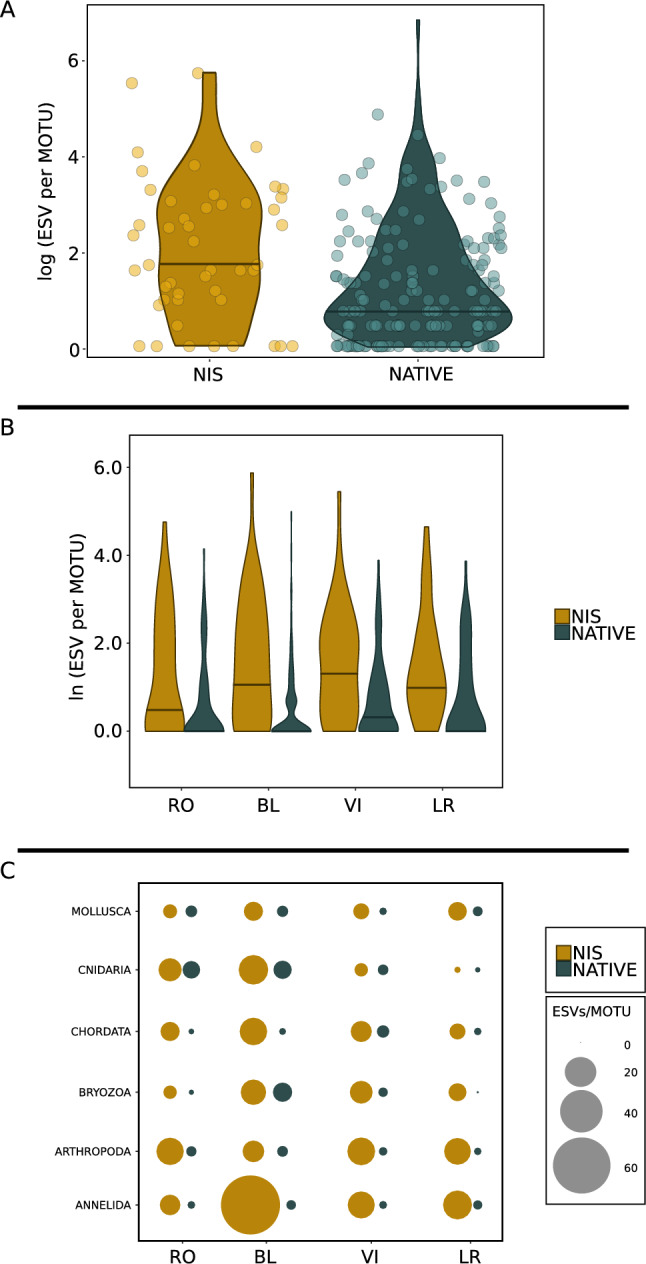
(**A**) Violin plots of the mean proportion of ESV per MOTU for NIS and native species. (**B**) violin plots separated by locality. (**C**) bubble plots as per phyla at each locality. Note natural logarithms in y axes of (**A**) and (**B**).

Figure. 7A shows the distribution of the number of ESVs per MOTU (log-transformed) in the NIS and NAT datasets. The 68% of the NIS MOTUs had some genetic variation, i.e., more than one ESV. This percentage was lower (47.5%) for the NAT dataset. In addition, the NIS with no genetic variation were low-abundance MOTUs (range of reads from 5 to 3,689), while NAT MOTUs with relatively high abundance can have no genetic variation (range of reads 5–23,591). The median number of ESVs per MOTU was 7.39 for the NIS and 2.72 for the NAT MOTUs (average of all samples), and the difference was significant (Mann-Whitney test, p<0.001). The number of ESVs per MOTU was also significantly higher in the NIS dataset when the data were separated by locality (Fig. 7B, Mann-Whitney tests, all p<0.003). A higher ESV abundance in NIS MOTUs was detected also when the datasets were divided by phylum (Fig. 7C).

We assessed the potential confounding effect of a relationship between the number of ESVs and number of reads in the MOTUs. There was indeed a significant correlation between the two variables (log-transformed) with *r*=0.853 and *r*=0.822 for NIS and NAT, respectively (Supplementary Material Appendix A, Fig A_1), and the slope of the regression was significantly higher for NIS. In the presence of these correlations, and to make values comparable, we performed a rarefaction of the number of reads of both NIS and NAT MOTUs to the mean read number of the NAT MOTUs (36,758 reads, MOTUs exceeding this value were rarefied). This rarefaction resulted in the elimination of 16.8% of the NIS ESVs and 2.3% of the NAT ESVs. The difference in number of ESVs per MOTU between NIS and NAT remained significant (Mann-Whitney test, p<0.001, Supplementary Material Appendix A, Fig A_2). A more stringent rarefaction to the median of the number of reads in the NAT dataset (354 reads) resulted in a marked elimination of 87.0% and 36.0% of the NIS and NAT ESVs, respectively. Notwithstanding, the ESV richness per MOTU remained significantly higher for NIS (Mann-Whitney test, p<0.001, Supplementary Material Appendix A, Fig A_3). These outcomes were not influenced by the different number of points (80 for NIS and 235 for NAT after outlier deletion), as shown by randomization tests resampling 100 times the NAT datasets to a size of 80 (Supplementary Material Appendix A). The result of a higher genetic diversity in NIS was therefore deemed robust.

**Fig. 8:**
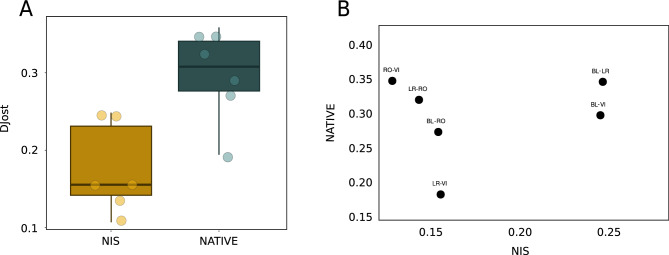
(**A**) Box-plots of the mean values of Jost’s D between localities for NIS and COMM datasets. Horizontal lines are medians, boxes encompass the first and third quartiles, whiskers indicate 10th and 90th percentiles. (**B**) Correlation plot for Djost for NIS and COMM datasets.

We obtained mean genetic differentiation (D) values between pairs of localities by averaging the D values of all MOTUs shared by each pair. These values (Fig. 8A) showed a lower differentiation in non-indigenous MOTUs (mean D=0.195) than in native MOTUs (mean D=0.314), and the difference was significant (paired-sample *t*-test, p=0.005). When the genetic distance measures for NAT and NIS were compared for each pair of localities (Fig. 8B), there was no significant correlation (*r*=0.19, p=0.6), indicating different patterns of genetic differentiation in both datasets.

### Comparison with a reference external point

Comparative analyses between the samples obtained inside (BL) and at a natural point outside (BO) the port of Blanes are presented in the Supplementary Material, Appendix B. The complete dataset for these two sampling spots is detailed in Table S5.

A total of 1,582 MOTUs and 30,351,305 reads were detected, of which 51 were assigned to NIS and 265 to NAT. For COMM, 732 MOTUs were exclusive to the natural community outside the harbour, and only ca. 18% of the detected MOTUs were shared between the two zones (Supplementary Material, Appendix B, Fig. B_1). For NIS, however, the proportion of shared MOTUs increased to ca. 55%, and only 7 MOTUs were detected exclusively outside, while 16 were found only inside.

A high proportion of reads assigned to NIS with respect to the total reads was recorded in BL (46%), in contrast to BO (11%). The proportions in terms of MOTU numbers were lower, although similar trends were observed, with a higher number of NIS detected inside the harbour (Supplementary Material, Appendix B, Fig. B_2).

The BO samples contributed a large number of MOTUs, which was reflected in significantly higher Shannon diversity and MOTU richness values compared with BL. In contrast, the NIS community showed significantly higher values inside the harbour for both the Shannon index and MOTU richness (Supplementary Material, Appendix B, Fig. B_3). Overall, the NIS were more abundant inside the harbour than outside it, and only six nominal species, assigned to seven MOTUs, exhibited a higher proportion of reads in BO compared with BL (Supplementary Material, Appendix B, Fig. B_4).

Both COMM and NIS assemblages showed a strong structuring between BL and BO. Bray–Curtis dissimilarity values, represented by NMDS ordinations, revealed a clear differentiation between the two zones, albeit more marked for the COMM than the NIS datasets (Supplementary Material, Appendix B, Fig. B_5).

In terms of intraspecific diversity, NIS exhibited a significantly higher number of ESVs per MOTU compared with native species inside the port (Supplementary Material, Appendix B, Fig. B_6). Conversely, outside the port, there was a significantly higher genetic diversity for the NAT assemblage. When the values were examined by phylum, this shift in the trend was also apparent, with a particularly high haplotypic diversity for annelids in BL and for cnidarians in BO. When we examined the MOTUs that were found both inside and outside for the NIS and NAT groups (28 and 76 MOTUs, respectively), the number of ESVs per MOTU was significantly higher inside the port for NIS, and outside the port for NAT MOTUs (Supplementary Material, Appendix B, Fig. B_7).

## Discussion

Our study showed that non-indigenous species exhibited higher population connectivity (considering both beta-diversity and genetic differentiation) and intraspecific genetic diversity compared to native species. These results support our initial expectation that maritime activities enhance NIS dispersal. The levels of genetic diversity detected in NIS suggests that the presence of high propagule pressure in ports contributes to their invasion success, fostering higher genetic variation within these artificial communities. In contrast, native species display more limited population connectivity and lower intraspecific genetic diversity, reflecting their higher isolation and vulnerability to impacts such as species invasions. These findings confirm that ports facilitate NIS’ range expansions, while the dispersal of native species among ports remains limited. By adopting a community-level approach and comparing native and NIS, our study provides valuable insights into population connectivity dynamics in modified ecosystems, an area that remains underexplored in the scientific literature, and provides a valuable baseline for future comparative studies.

DNA metabarcoding data from different substrates (water, settlement plates, sediment) and genetic markers offer different windows on port biodiversity, and combining them is recommended for detailed studies^40,41,63^. For broad-scale spatio-temporal comparisons of metazoan diversity, a cost-effective and practical approach is to use a standardised method such as the POMPOM collectors^64^. Rarefaction curves indicated that our sequencing depth was sufficient to capture the diversity in each sample, although accumulation curves suggest that additional replicates could have revealed rare MOTUs, something common in metabarcoding studies^65–67^. Thus, we detected a substantial proportion of the biodiversity present in the studied ports that can be used to confidently assess patterns of population connectivity and intraspecific genetic diversity.

Interestingly, while NIS represented less than 4% of the total MOTUs, they had a disproportionate abundance in terms of the number of reads, comprising between 34 and 70% of the reads in the port samples. In another metabarcoding study of harbour communities, Lavrador et al. 2024^40^ detected a similar percentage in species richness (3.6%) of NIS using several substrate types. Arthropods, cnidarians, and chordates (ascidians) dominated port communities in our samples, and a gradient was detected as the abundance of cnidarians decreased, and that of arthropods increased from north to south. No similar gradient was observed in the taxonomic composition of the NIS found in the ports, dominated by arthropods, annelids and chordates. There were instances of several MOTUs being assigned to the same nominal species. This happened more frequently in NIS (57 nominal species for 82 MOTUs) than in NAT MOTUs (213 nominal species for 237 MOTUs). This can be attributed to the presence of species complexes, more commonly reported in the broadly distributed NIS. We have therefore performed most analyses on MOTUs and not nominal species.

The southernmost ports (VI and LR) displayed an overall lower MOTU richness, and LR had the highest abundance and diversity of NIS. For LR, NIS accounted for 70% of the total reads, with three MOTUs dominating (55% of the total reads), assigned to the amphipods *Elasmopus rapax* and *Laticorophium baconi*, and the isopod *Paracerceis sculpta*. It is unclear which factor or combination of factors can drive these differences. Temperature may play a role, as readings indicated a warmer summer, but colder winter, in the southernmost site. In addition, our sampling points are interspersed with two of the largest commercial ports in the Western Mediterranean, namely Barcelona (between BL and VI) and Tarragona (between VI and LR). These large harbours are home of intense international cruise and cargo trade operations and act as entry points for species and subsequently influence the composition of nearby smaller ports via local boating. However, the higher presence of NIS in LR is more likely explained by the proximity of this port to the lagoons of the Ebro Delta, one of the largest bivalve aquaculture regions of the Mediterranean Sea. Indeed, the Ebro Delta area is a NIS hotspot^68^ and can thus influence the species composition of surrounding areas.

Metrics derived from relative read abundances are commonly used in metabarcoding studies, yet they can be affected by several sources of bias and therefore should not be regarded as a direct proxy for metazoan biomass. Nevertheless, some studies have reported a general relationship between relative biomass and read proportions in COI metabarcoding datasets (e.g., zooplankton^69^:; epibenthos^70)^:. Moreover, the primer pair employed here has been shown to provide useful quantitative information^71^. On this basis, we consider that our dataset contains information beyond simple presence–absence patterns, and we have therefore included relative read frequencies in our analyses. In any case, assuming that potential biases are consistent across samples, the comparative interpretation of the results should remain reliable^72^.

Our NMDS configurations for overall community composition in ports showed a north to south gradient. Interestingly, all four ports clustered separately (with some overlap) in the final configuration, highlighting significant spatial structure at this relatively small scale. In line with this, the NMDS analysis of the MOTUs identified as NIS indicated a similar overall trend, but with a markedly higher overlap and less distinctness of the communities in each locality studied. The finding of weaker spatial structure in the MOTUs assigned to NIS, compared to the overall community, was consistent with the greater MOTU sharing observed in the NIS dataset, as shown by a significantly higher Sørensen index. In fact, 36.6% of the NIS MOTUs were detected in all study areas, whereas 66.7% of the MOTUs in the COMM dataset were restricted to only one of the four localities. This contrasts with the 6.4% of COMM MOTUs that were found across all four localities. Boat traffic likely fuels this differential connectivity of species well adapted to travel as hitchhikers on hulls^19^. For most other species, coastal drift or other natural dispersal processes are the only way to ensure some degree of connectivity^1^. Whether the shared diversity observed in NIS is due to exchanges between the network of small ports in the area^62^ or to repeated introductions from introduction hubs (big ports or aquaculture facilities) could not be determined, although the small size of the ports points to local boating exchanges as the potential main vector.

We identified some interesting temporal patterns in our samples, such as the quantitative replacement of some groups over the studied months, both for NIS and for the wider community. Common NIS occurring along the Catalan coast are known to show highly seasonal patterns^73^, while other NIS are present and reproductive all year around^74^. We also detected differences in beta-diversity analyses, such as clustering in cold or warm periods of the samples. Previous metabarcoding studies have also detected seasonal changes in port communities^40,41^.

The use of a denoising method customised for coding sequences to generate an ESV dataset allowed us to uncover intra-MOTU patterns. In this context, we used ESVs as a proxy for haplotypes in the same way that MOTUs are a proxy for species. To ensure a more targeted comparison, we selected a subset of MOTUs from the COMM dataset that could be confidently assigned to native species. We focused our analyses on these native MOTUs rather than the rest of the community, as MOTUs not identified at species level may include cryptogenic or unrecognised NIS, potentially biasing intraspecific diversity estimates. We then examined their composition in terms of ESVs within MOTUs as compared with the NIS dataset. This comparison allowed an assessment of the scope for adaptation of these groups. A first analysis showed that the genetic richness was significantly higher in NIS compared to NAT. In addition, this holds true on a phylum-by-phylum basis, showing that this effect is not restricted to a particular group behaving differently. The pattern remains significant when MOTUs are rarefied to avoid any confounding effect of the different numbers of reads. This result was somewhat unexpected, as NIS are supposed to experience bottlenecks when they colonise new areas. The so-called genetic invasion paradox^50^ has long been a matter of debate, as NIS often thrive even if their genetic variability is supposedly drastically reduced. This reduction may simply be a wrong assumption, as recurrent introduction of propagules from different regions can in fact lead to higher genetic diversity in introduced than in natural populations of these species, thus solving the paradox^17,48,50^. In contrast, native species may rely on a more limited source of new propagules in ports^75^ and may become comparatively haplotype-poor. Finding suitable environments combined with high genetic diversity may be key to their successful settlement, survival, and dispersal strategies^50^, which is key for eventually becoming a biological invasion problem^76^. Genomic features, such as introgressions^77^, inversions^78^, or the microbiome^45,79^ have been proposed as possible explanations for the fast adaptation of NIS to new environments, and a high genetic variability can also contribute to their ability to do so.

Our finding of high genetic connectivity observed among non-indigenous species in Mediterranean ports showed evidence of intense gene flow across multiple locations, contrasting with the more marked spatial structure observed in native species. This high level of connectivity likely promotes genetic homogenisation and helps maintain elevated intraspecific diversity, which is known to enhance the adaptive capacity and invasive success of NIS. As seen in previous studies, our results confirm the role of ports as networks of propagation of NIS^62^ and the existence of a portuarization syndrome^11,12^, whereby ports act as replicate natural laboratories, favouring the evolution of well-adapted, homogenised NIS biota. In this context, the comparison carried out in the Blanes area between samples collected inside and outside the port provides additional evidence for this process. While the natural communities outside the harbour showed higher relative abundance and higher genetic diversity values for native species, NIS exhibited greater relative abundance and genetic diversity within the harbour. Furthermore, only a small fraction of the total MOTUs was shared between the two zones, confirming that port environments host biological assemblages that are distinct from those of the adjacent natural habitats. These results support the idea that ports act as stepping-stone habitats that particularly favour the establishment and proliferation of non-indigenous species, reinforcing the process of biological homogenisation associated with maritime activity. Although the data derive from a single locality, they clearly reinforce the idea that port NIS stocks are not supplied from nearby natural habitats, but by interchanges with other ports via boating. This phenomenon not only fuels biological invasions but also poses a critical challenge for the management and conservation of coastal ecosystems, as high genetic diversity and high adaptive capabilities in NIS may increase their resilience to environmental changes and their potential to spread and displace native communities.

## Materials and methods

### Sampling and sample processing

We focused on the Catalan coastline (NW Mediterranean), a densely urbanised region with a high concentration of ports^62^. We used a recently designed sampling device called POMPOMs^64^, which consists of a 100 × 25 cm strip of polyamide mesh with hexagonal openings of approximately 1 mm, folded in a zig-zag pattern and fastened with cable ties to form a circular shape (approximately 25 cm in diameter). POMPOMs are capable of capturing both early life-history stages and particulate organic matter, thereby providing a comprehensive snapshot of the biodiversity present.

POMPOMs were placed underwater (ca. 1 m deep) hanging from dedicated ropes in the ports. Four replicate collectors were deployed in four ports on the Catalan coast (northwestern Mediterranean): Roses (42.254495 °N, 3.180719 °E), Blanes (41.674425 °N, 2.799687 °E), Vilanova i La Geltrú (41.214873 °N, 1.736144 °E), and La Ràpita (40.618726 °N, 0.598968 °E) (see details in Fig. S7). We will hereafter denote these locations as RO (Roses), BL (Blanes), VI (Vilanova I la Geltrú), and LR (La Ràpita). All these ports are medium-sized and sustain fishing and recreational boating activity. They comprise altogether between 1,500 and 3,000 lineal metres of docks. The distance by sea between the two most distant ones (RO and LR) is 323 Km. In addition, we also sampled as a reference point a natural rocky reef at 4 m depth (41.673020 ºN, 2.803294 ºE) outside the Blanes port (ca. 700 from the mouth), hereafter BO (Blanes outside the port), in order to assess the potential effect of the nearby natural community on the port assemblages. POMPOMs in this rocky reef were tied to ropes anchored to the bottom and held vertically by small floats at a depth equivalent to the POMPOMs placed inside the ports.

The POMPOMs were deployed from February 2019 to April 2020. They were replaced monthly during warm months and bimonthly during cold months, resulting in a total of 10 temporal sampling points. Three collectors per month and site were used for metabarcoding studies, while the fourth was retained as a backup. In total, 120 samples were obtained for this study. After collection, the POMPOMs were unfolded, and all biofouling was carefully removed using sterilised 10 cm nylon brushes and recovered in a stainless-steel sieve of 64 µm.

We conducted seawater temperature readings at each site every hour over the study period using HOBO^(R)^ Pendant data loggers (resolution 0.53ºC), which were placed right next to the POMPOMs (Fig. S5).

### DNA extraction & sequencing

All procedures were performed in a sterilised laminar flow cabinet, with UV light activated between each sample processing. We followed the DNA extraction, library preparation, and sequencing protocols detailed in^80^. In short, DNA was extracted from 5 g of homogenised sample material using the DNeasy PowerMax Soil Kit (Qiagen). A fragment of ca. 313 bp of the cytochrome c oxidase subunit I (COI) gene was amplified using as generalist primers for eukaryotes the Leray-XT primer set^67^, which includes the forward primer mlCOIintF-XT: 5’-GGWACWRGWTGRACWITITAYCCYCC-3’ and the reverse primer jgHCO2198: 5’-TAIACYTCIGGRTGICCRAARAAYCA-3’^81^. Both primers were tagged with an 8-base sequence at the 5’ end, with distinct tags for each sample differing by at least three bases. The same tag was used for both forward and reverse primers of each sample to facilitate the elimination of inter-sample chimeras. In order to enhance sequence diversity and facilitate Illumina base calling, a variable number of degenerate bases (N), from two to four, were added before the tags on both primers. The PCR mix for each sample consisted of 10 µL of Amplitaq Gold 360 Master Mix, 0.16 µL of BSA, 5.84 µL of H₂O, 1 µL of each primer (forward and reverse) at a concentration of 5 µM, and 2 µL of DNA. The PCR procedure consisted of a first denaturation step for 10 min at 95ºC, followed by 45 cycles of denaturation at 94ºC for 60 s, hybridisation at 45ºC for 60 s, and elongation at 72ºC for 60 s, ending with a final elongation step of 5 min. The amplification products were then purified and concentrated using the MinElute PCR Purification Kit (Qiagen). The success of the amplification was verified using gel electrophoresis. Negative samples (n=12) were run by processing and extracting samples of sand charred in a muffle furnace. Fifteen PCR blanks, containing no DNA template, were also included. Libraries were prepared using the BIOO NEXTFLEX PCR-Free DNA-Seq Kit (Perkin-Elmer) and sequenced on a partial Illumina NovaSeq lane with 2 x 250 bp paired-end sequencing at Novogene Company.

### Bioinformatic analyses

We used a custom pipeline based on the Obitools3 software^82^ and using both Bash and R 4.0.2 scripts. Briefly, Illuminapairedend was used to align paired-end reads, retaining only those reads with an alignment quality score greater than 40. Reads were then demultiplexed using ngsfilter, discarding any reads with unmatched primer tags at their ends. The obigrep and obiuniq functions were used to perform a length filter (retaining only sequences between 310 and 319 bp) and combine identical sequences. Singleton sequences (with just one read) were deleted at this step. The Uchime de novo algorithm of VSEARCH v2.7.1 was then used to remove chimeric amplicons^83^. Before any filter, the control samples had 352,481 reads, while collector samples had 219,821,103 reads.

Sequences were denoised with the DnoisE program^54^, which is a modification of the Unoise algorithm^84^ that incorporates the natural variability in the three codon positions of coding genes. Denoising was performed within samples with an alpha parameter of 4 and an auto-computed entropy correction to generate Exact Sequence Variants (ESVs)^53^. The following filters were then applied to the ESV dataset: We first deleted any ESV for which the sum of all its reads in blanks or negative controls represented more than 10% of the total reads for that ESV in all samples, as these are suspected to correspond to contaminations. Second, for each sample, a dual abundance filtering was established, setting to zero the reads of (i) ESVs that represented less than 0.005% of the sample total reads, and (ii) ESVs with less than 5 reads after the previous step. The 0.005% value has been selected to approximate thresholds found in previous simulations and analyses using changes in entropy values to control the elimination of errors^52,54^. A similar optimal value was determined by^85^ comparing different thresholds for COI, so it may represent a good compromise between eliminating errors and keeping legitimate, but rare, sequences. This same filter has been applied previously to metabarcoding datasets using this marker^53,86,87^.

ESVs were then clustered into molecular operational taxonomic units (MOTUs) with SWARM v3.1.3 using d=13, following^53^. SWARM is a variable-threshold and fast algorithm that connects all reads with a distance less than d in a first step and then breaks down the resulting clusters using a topological criterion based on the internal abundance structures of the clusters^88,89^. The most abundant ESV in the MOTU was used as the representative sequence. The information of which MOTU each ESV was assigned to was also extracted from the SWARM output and added to the ESV dataset.

Taxonomic assignment of the representative sequences of the MOTUs was performed using the mkLTG software^90^ with the COInr database^91^. The default parameters of the mkLTG procedure were slightly modified (available at https://github.com/jesuszarcero/mkLTG_params_modified). To avoid overclassification^92^, we eliminated Insecta and Arachnida (but keeping Acari) from the reference database using the mkCOInr program^91^. Only MOTUs assigned to Metazoa were kept for downstream analyses. The final dataset refinement consisted of applying to the MOTUs dataset the LULU post-clustering correction procedure^93^. This procedure combines similarity and co-occurrence metrics to detect erroneous MOTUs and pools the reads with the correct ones. The ESV dataset was updated accordingly. We used a modification of the original LULU function (https://github.com/jesuszarcero/LULU_corrected), which solves a known issue in the original script(https://github.com/tobiasgf/lulu/issues/8), whereby the program fails to merge some MOTUs when relative cooccurrence parameter is set to less than 1. A final checking of erroneous sequences, including nuclear mitochondrial inserts (numts) was performed following^52^ and^64^: all ESVs whose representative sequences had codon stops were deleted, after checking the 12 metazoan mitochondrial genetic codes from the Biostrings R package^94^. We also checked the five amino acids conserved across metazoans in the studied fragment^95^, and sequences with changes in these positions were deemed as incorrect and the corresponding ESV deleted. If all ESVs of a MOTU were eliminated, the latter was also deleted from the final MOTU table.

NIS detection was performed using the NCBI-BLAST algorithm^96^ and the custom NISdb v3 database^64^ (https://github.com/jesuszarcero/NISdb), which contains curated sequences of NIS found in the Mediterranean Sea. The NIS database was compiled based on the available literature on NIS in the Mediterranean and is regularly updated. Subsequently, we searched for available COI-5P sequences of these species in the Barcode of Life Data System (BOLD, https://www.boldsystems.org/). Whenever NIS sequences were not found in BOLD, we referred to the NCBI database and verified species identification using the taxonomic literature. For each NIS, the obtained sequences were collapsed into unique haplotypes. To ensure data accuracy, a thorough manual curation was conducted, involving comprehensive BLAST searches and the removal of potentially erroneous sequences whenever the BLAST results indicated a different species or conflicting assignments. BLAST results were filtered, retaining assignments with an identity equal to or greater than 97% and a sequence coverage equal to or greater than 70%. Assignments against this database were compared with the corresponding MOTU assignments obtained with the mkLTG procedure to check the accuracy of NIS detection with the general database.

### Datasets

Downstream analyses were conducted using three different datasets with the port samples, depending on the objective of each analysis. We first identified MOTUs assigned to non-indigenous species (NIS) following the procedure described above, and the remainder of the community (hereafter the COMM dataset). This dataset includes many MOTUs that could not be identified to species level; therefore, their exact status as native or NIS could not be determined. Genetic databases are likely to provide better coverage of NIS than of native species^97^, as the former are more extensively studied due to their socio-economic impacts. This likely inflates the apparent diversity of NIS. Consequently, we assume that most COMM MOTUs lacking species-level assignment are likely native. However, to enable more focused comparisons with the NIS dataset, we also selected those COMM MOTUs identified to species level and confidently assigned as native (hereafter NAT). The NAT list was manually curated to exclude problematic MOTUs, such as species complexes, dubious database matches, or cryptogenic species.

Additionally, a dataset was created with just the samples from inside and outside the Blanes port (BL and BO), to allow for comparisons between this port and the outside reference point.

### Statistical analyses

Unless otherwise stated, analyses were conducted with the ‘vegan’ R package^98^, and plots were created with the ‘ggplot2’ R package^99^. Rarefaction curves and species accumulation curves were generated with the functions rarecurve and specaccum, respectively. We considered that a plateau was reached in these curves if the slope fell below 0.01. MOTU richness and Shannon diversity values were computed after rarefaction to the minimum number of reads in any sample using the function rarefy. The resulting metrics were compared across categories with ANOVAs and post hoc Tukey tests for Shannon diversity, and Kruskal-Wallis followed by post hoc Dunn tests for MOTU richness (as assumptions for parametric tests did not hold). Likewise, these metrics were compared in the samples inside and outside the Blanes port with non-parametric Mann-Whitney tests.

To assess the taxonomic composition of the samples, MOTUs were grouped into major metazoan phyla, and bar charts were generated to display the average composition per locality in terms of proportion of reads and proportion of MOTUs for both the COMM and the NIS datasets. The proportion of reads assigned to each phylum was also broken down by month at each locality to ascertain temporal trends. Additionally, a heatmap was constructed to visualise the prevalence of each NIS across ports. The presence-absence based Sørensen index between pairs of samples was computed to reveal patterns of MOTU sharing across localities for the COMM and NIS datasets, and these patterns were further visualised by upset plots.

For β-diversity analysis, we used the relative read abundance of each MOTU in each sample without rarefaction and computed the Bray-Curtis dissimilarity index (BC). These values were then used to generate reduced-space representations of the samples in a non-metric multidimensional scaling (NMDS) configuration using the metaMDS function of vegan, separately for COMM and NIS MOTUs. We also generated NMDS plots for the dataset including the samples from inside and outside the Blanes port.

Permutational analyses of variance (PERMANOVA) were conducted for the factors site and month on the BC matrix using the PERMANOVA module of the statistical package Primer v6^100^. Pairwise tests for significant factors were performed. A total of 999 permutations were used to generate the statistics of these analyses. We also performed dispersion tests (permdisp procedure with 999 permutations) to assess differences in multivariate dispersion of the groups of samples. Finally, we analysed temporal trends of community change by averaging Bray-Curtis dissimilarities among consecutive sampling times, separately for the COMM and NIS datasets.

### Metaphylogeographic analyses

We also performed analyses of genetic diversity within MOTUs (our proxy for species-level diversity) using the ESVs as a proxy for their haplotype composition^52^. For this assessment, we restricted our analyses to comparisons of the NIS and NAT datasets to ensure a correct assignment to these categories, as the COMM dataset had many MOTUs unassigned at species level. We first obtained the values of ESV richness in NIS and NAT MOTUs and visualised them in violin plots. Given their highly skewed nature, they were compared via a non-parametric rank test (Mann-Whitney’s test). As the number of ESVs can be dependent on the read abundance of the MOTUs, we also computed regressions between both variables independently for NIS and NAT MOTUs, checked the homogeneity of the slopes, and rarefied the data to eliminate the confounding effect of read abundance. We performed a first rarefaction to the mean number of reads found in NAT MOTUs, in which the reads of all MOTUs (NIS or NAT datasets) exceeding this read number were rarefied to this common value, keeping the proportions of reads of each sample the same as in the original datasets. In a second, more stringent rarefaction we used the median of the read numbers found in NAT MOTUs. We repeated the Mann-Whitney tests for the rarefied datasets to check the results previously obtained.

Comparisons were also performed splitting the data per locality and per major Phylum to test if the patterns observed were general or driven by some local setting or taxonomic group. Finally, we also compared the ESV richness in Blanes for the NIS and native MOTUs inside and outside the port.

We then computed genetic differentiation measures among pairs of port localities using the D statistic^101^ with the function *pairwise_D* of the R package ‘mmod’^102^. We selected, for each pair of localities, those shared MOTUs with at least 2 ESVs in the localities in order to ensure a minimal level of genetic variation. Read frequency data do not provide a direct measure of abundance^52^, thus an indirect estimate was used, following^80^ and^103^. We considered as an abundance approximation the frequency of occurrence of each ESV, that is, the number of samples where each ESV has been detected in each locality. Occasional negative D values were transformed to zeros. These analyses were performed separately for MOTUs assigned to NIS and NAT, and Jost’s D values were compared among pairs of localities with paired-sample t-tests.

## Fundings

This research was funded by projects MARGECH (PID2020-118550RB) and BlueDNA (PID2023-146307OB) funded by the Spanish Ministry of Science, Innovation, and Universities (MICIU/AEI/10.13039/501100011033) and by ERDF/EU. J.Z. was funded by grant PRE2021-097703 (MICIU/AEI/10.13039/501100011033 and ESF+). Funding was also provided by the European Union (GA#101059915 - BIOcean5D), views and opinions expressed are those of the authors only and do not necessarily reflect those of the European Union. Neither the European Union nor the granting authority can be held responsible for them. AA was funded by the European Union’s Recovery and Resilience Facility-Next Generation, in the framework of the General Invitation of the Spanish Government’s public business entity Red.es to participate in talent attraction and retention programmes within Investment 4 of Component 19 of the Recovery, Transformation and Resilience Plan.

## Supplementary Information

Supplementary Information 1.

Supplementary Information 2.

Supplementary Information 3.

Supplementary Information 4.

Supplementary Information 5.

Supplementary Information 6.

Supplementary Information 7.

Supplementary Information 8.

Supplementary Information 9.

Supplementary Information 10.

Supplementary Information 11.

Supplementary Information 12.

Supplementary Information 13.

## Data Availability

All sequences generated were uploaded to the NCBI SRA archive: Bioproject PRJNA1179718.
