# Supplementary figures and images for: Spatio-temporal metabarcoding surveys in ports reveal homogenised communities of non-indigenous species with high genetic diversity and connectivity
Source: Sci Rep. 2026 Apr 26;16:15517. doi: 10.1038/s41598-026-49393-3 (PMC13187133; doi:10.1038/s41598-026-49393-3)

A

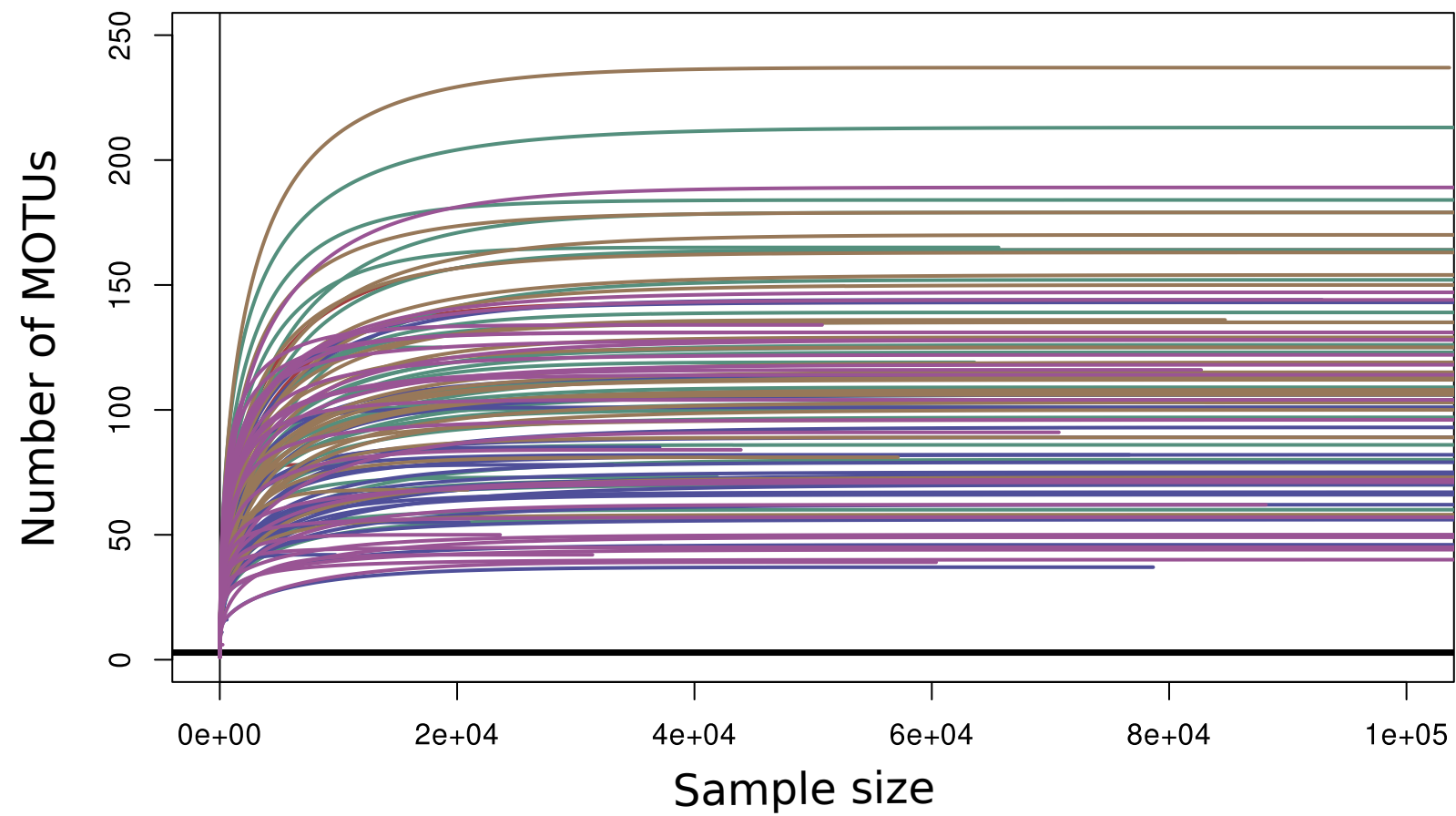

B

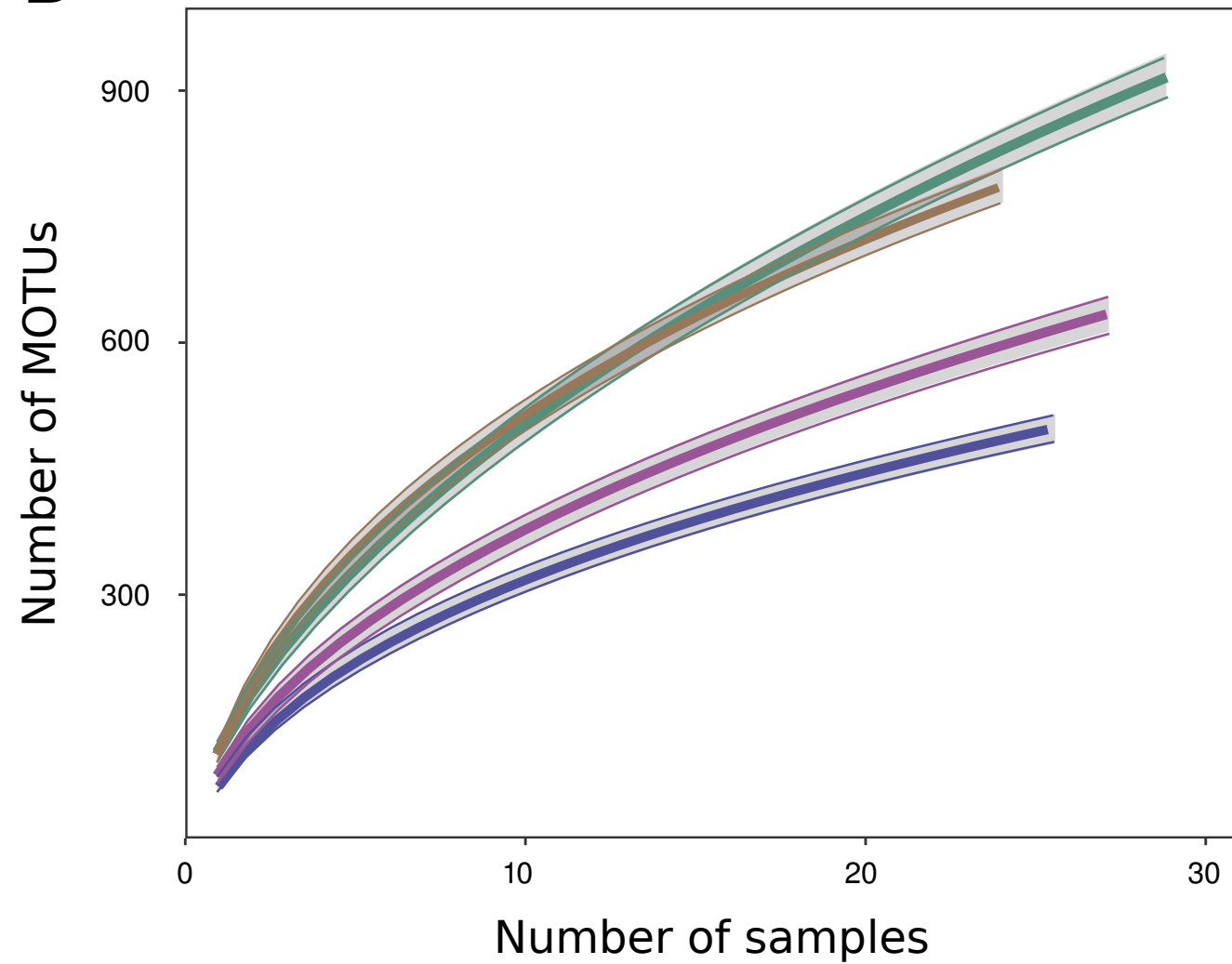

Supplement: Supplementary file 1 — Supplementary Information 1. [file 41598_2026_49393_MOESM1_ESM.pdf]

Proportion of COMM reads

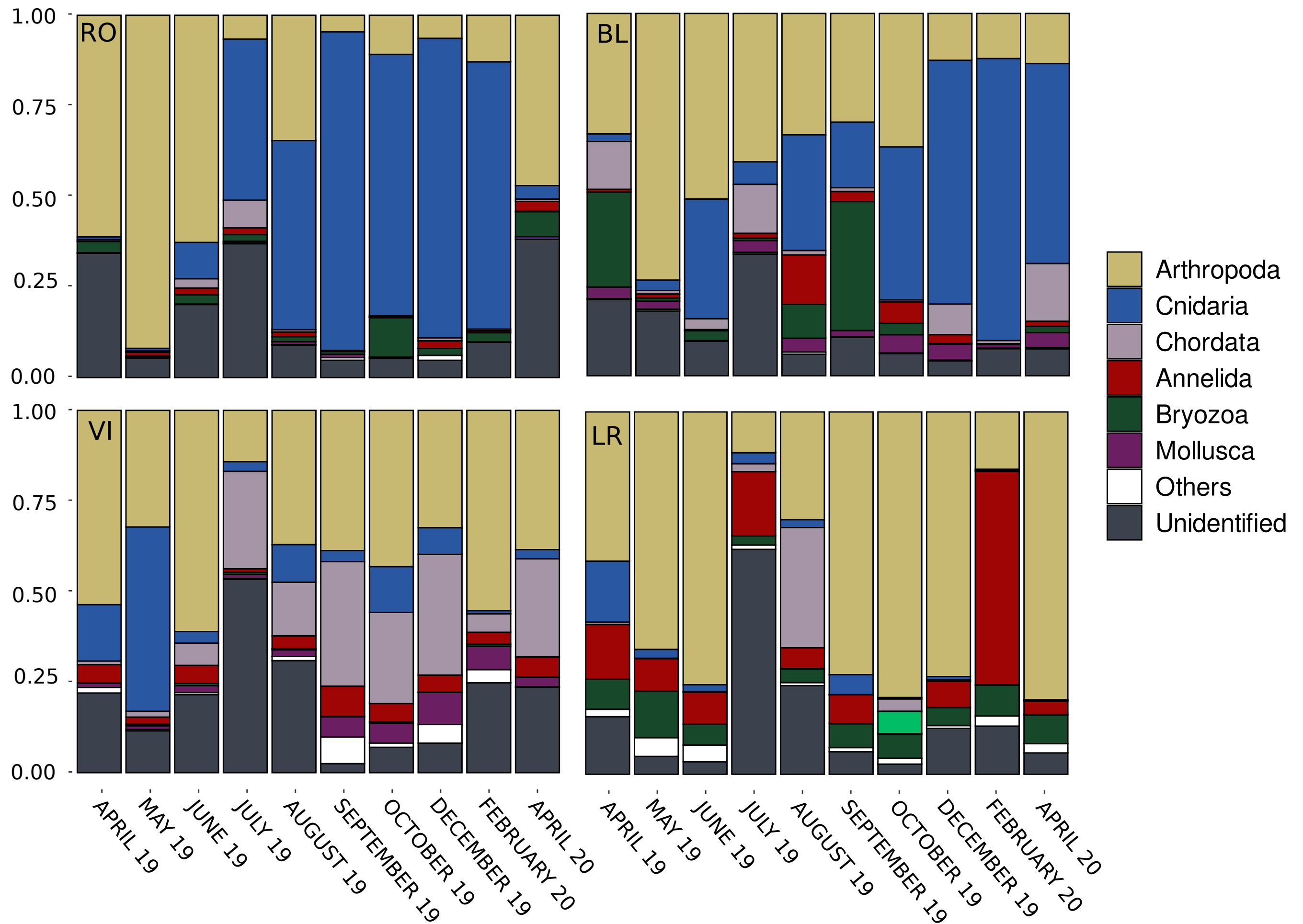

Supplement: Supplementary file 2 — Supplementary Information 2. [file 41598_2026_49393_MOESM2_ESM.pdf]

Proportion of NIS reads

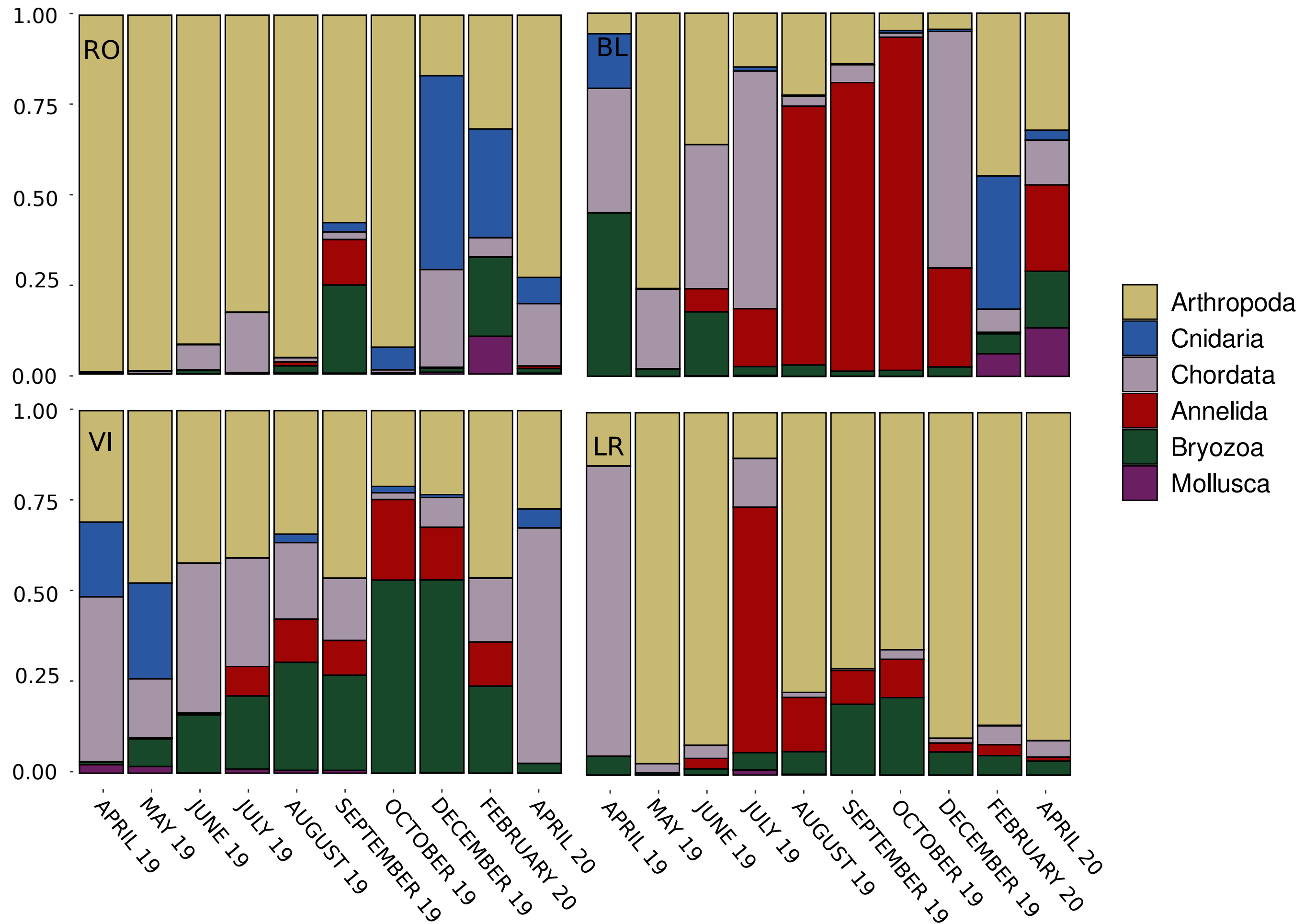

Supplement: Supplementary file 3 — Supplementary Information 3. [file 41598_2026_49393_MOESM3_ESM.pdf]

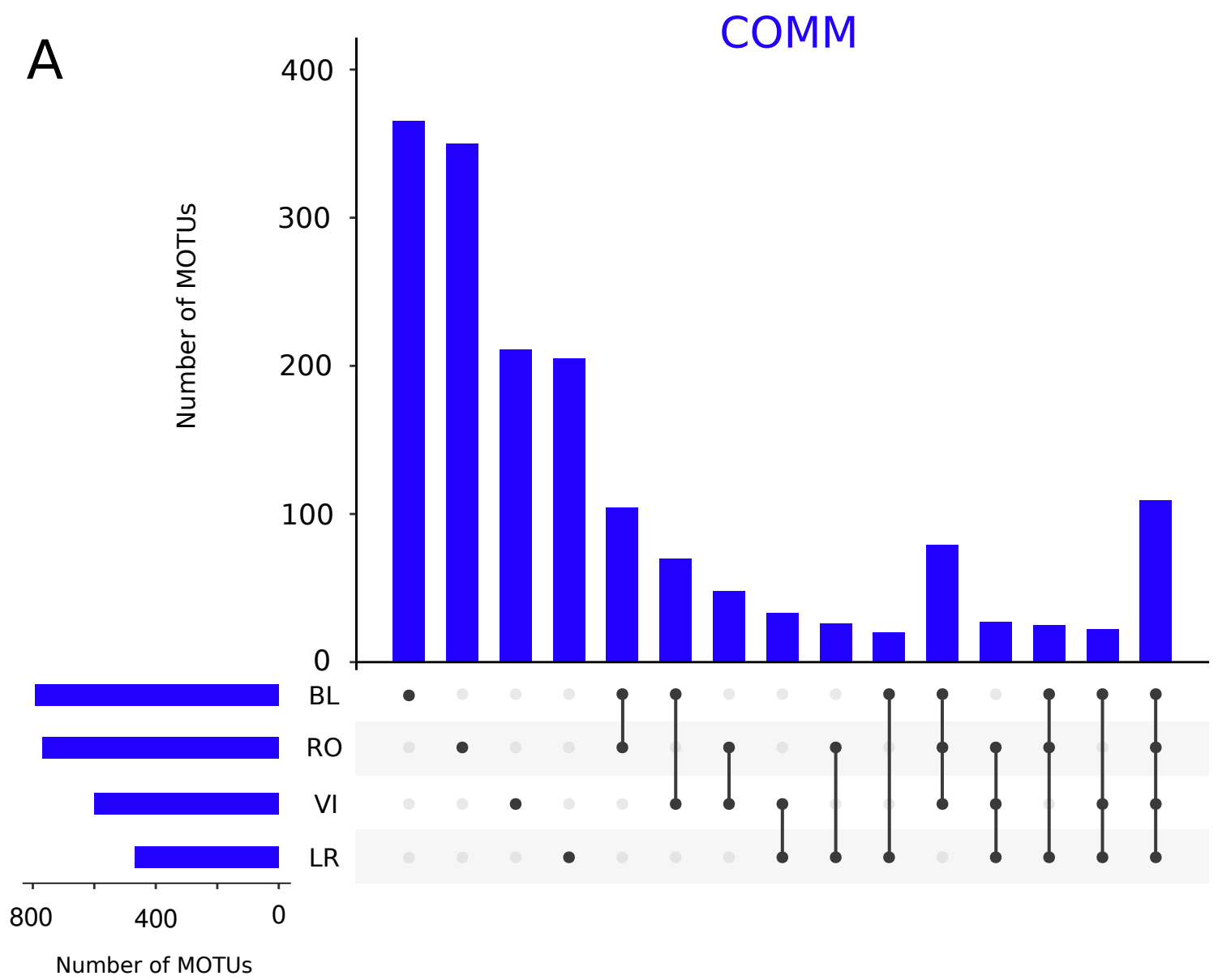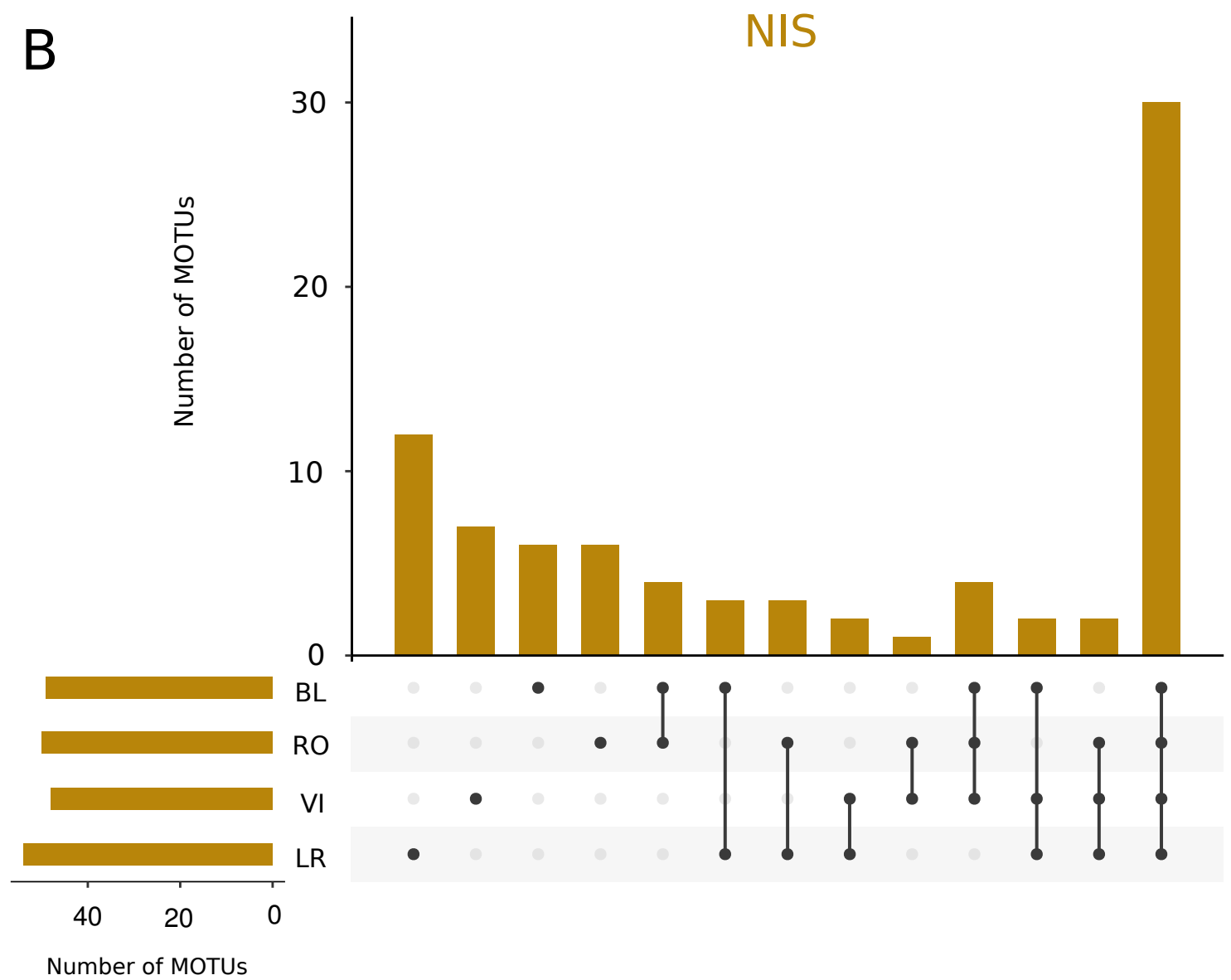

Supplement: Supplementary file 4 — Supplementary Information 4. [file 41598_2026_49393_MOESM4_ESM.pdf]

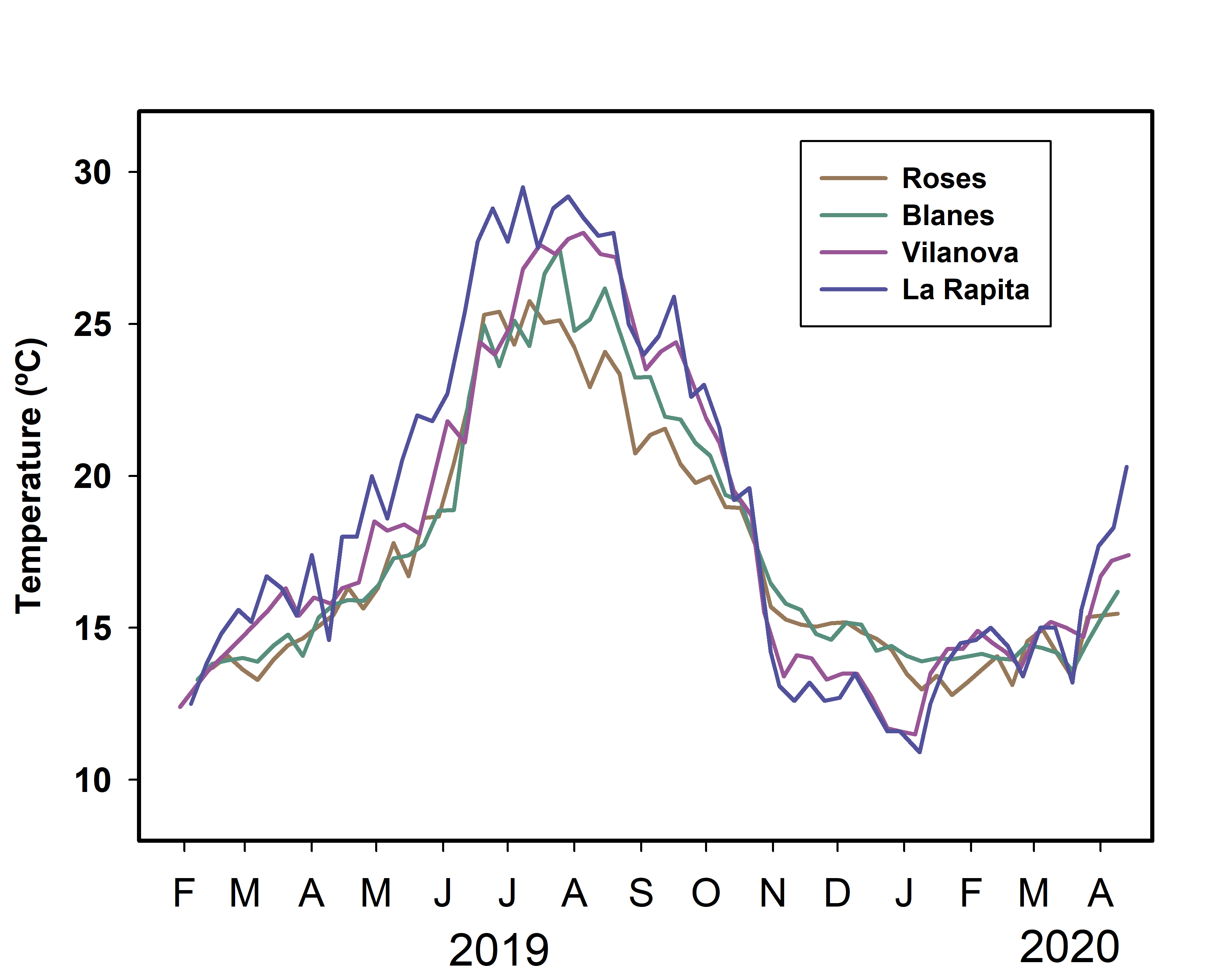

Supplement: Supplementary file 5 — Supplementary Information 5. [file 41598_2026_49393_MOESM5_ESM.jpg]

BC NIS

0.8

0.7

0.6

0.5

Apr 19-May 19

May 19-Jun 19

Jun 19-Jul 19

Jul 19-Aug 19

Aug 19-Sep 19

Sep 19-Oct 19

Oct 19-Dec 19

Dec 19-Feb 19

Feb 19-Apr 20

● COMM ● NIS

0.8

0.7

0.6

0.5

BC COMM

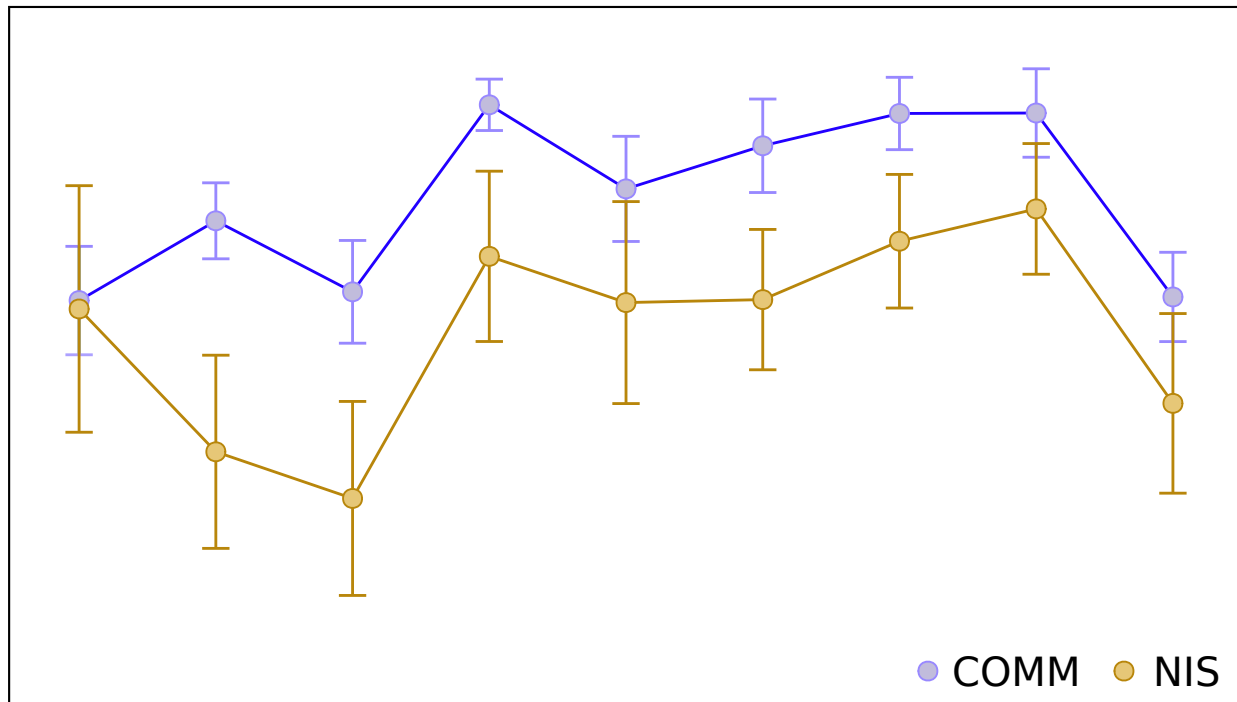

Supplement: Supplementary file 6 — Supplementary Information 6. [file 41598_2026_49393_MOESM6_ESM.pdf]

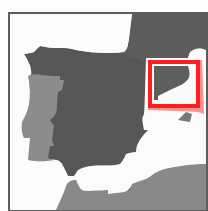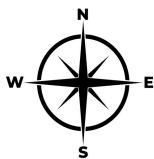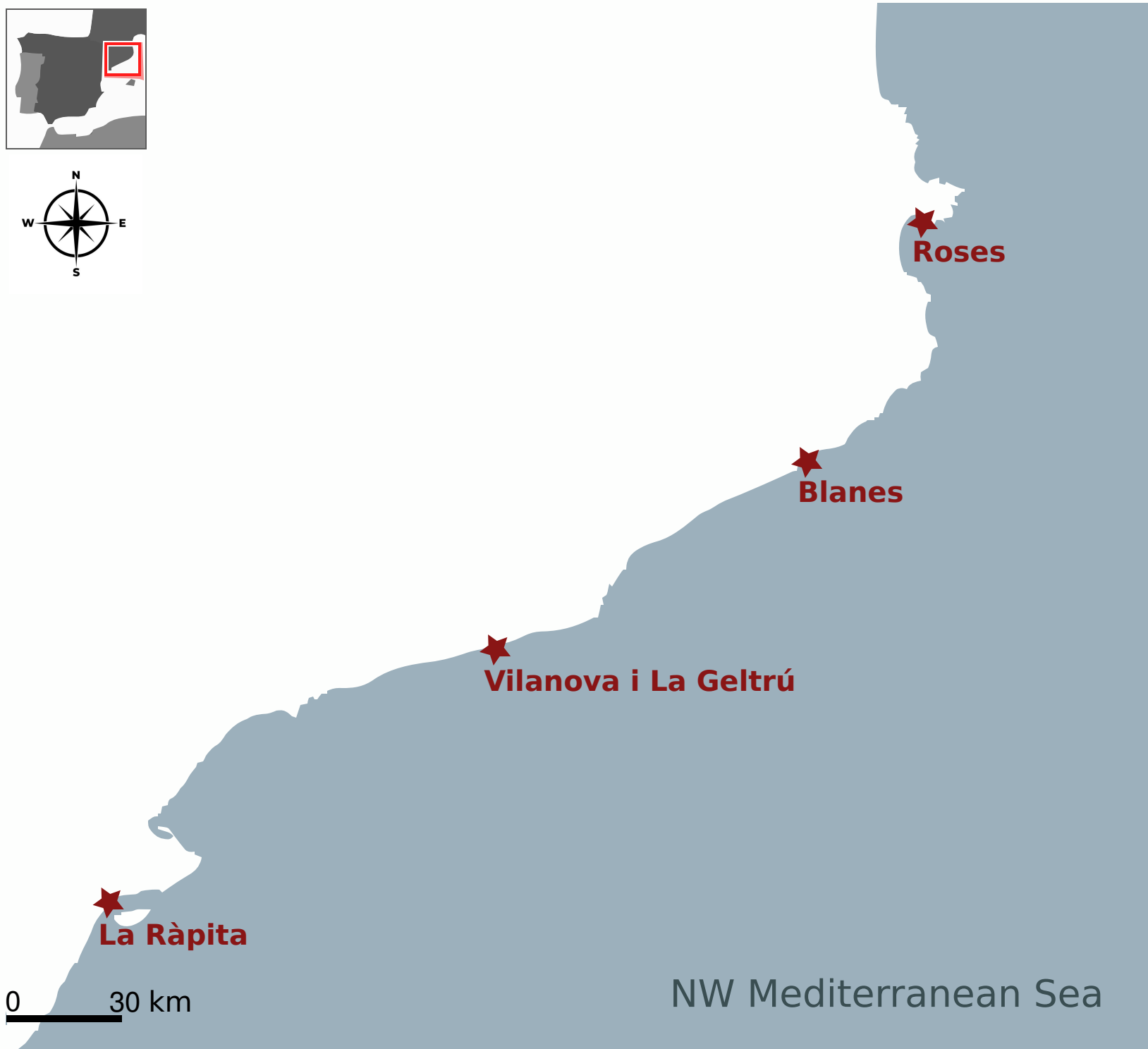

0 30 km

NW Mediterranean Sea

Supplement: Supplementary file 7 — Supplementary Information 7. [file 41598_2026_49393_MOESM7_ESM.pdf]
