# Supplementary material for: Spatio-temporal metabarcoding surveys in ports reveal homogenised communities of non-indigenous species with high genetic diversity and connectivity
Source: Sci Rep. 2026 Apr 26;16:15517. doi: 10.1038/s41598-026-49393-3 (PMC13187133; doi:10.1038/s41598-026-49393-3)
Supplement: Supplementary file 8 — Supplementary Information 8. [file 41598_2026_49393_MOESM8_ESM.pdf]

Appendix A- Rarefaction analyses

The NIS and NAT datasets were screened for the relationship between number of ESVs and number of reads. There were significant correlations of both variables (in log scale), with r=0.853 and r=0.822 for NIS and NAT, respectively (both p<0.001) (Fig. A_1). Note that two outliers (one for number of reads and another for the number of reads) were detected and removed from each dataset. The slopes of the regressions were 0.378 and 0.287 for NIS and NAT, respectively.

The homogeneity of slopes was assessed by fitting the model lm(log(n_ESVs)~log(n_reads)+group+group*log(n_reads))

Where group is a categorical variable with levels NIS and NAT. The interaction term of the model was significant (F=11.114, p<0.001) indicating that the slope of NIS was significantly higher than that of NAT.

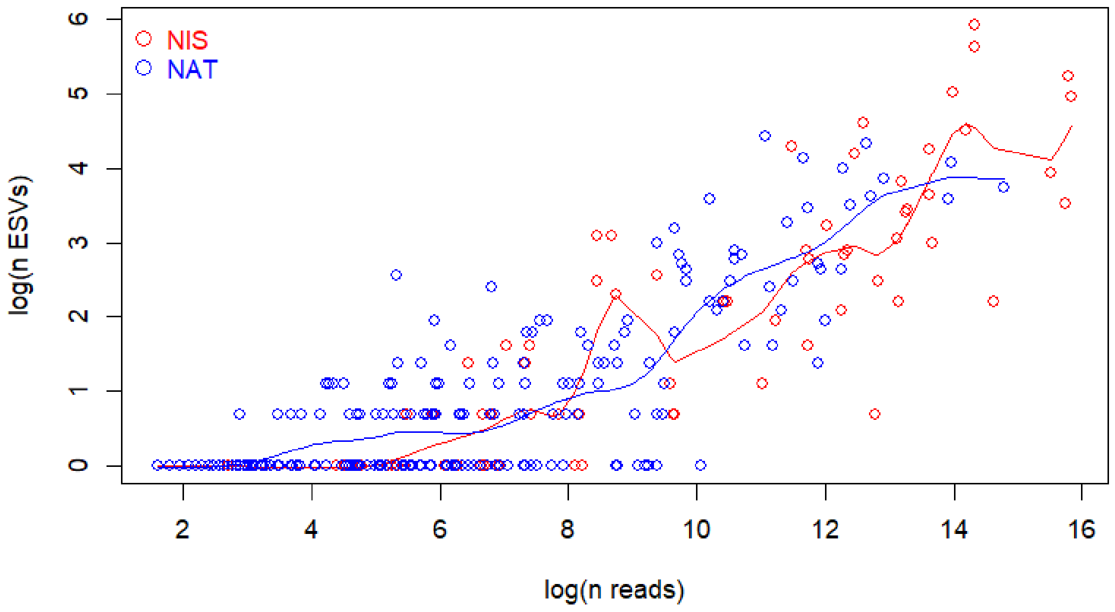

Fig. A_1. Scatter plots of the number of ESVs vs the number of reads (log transformed) for NIS (in red) and NAT (in blue) MOTUs. A cubic smoothing spline is added for each dataset.

In the absence of homogeneity of slopes, we rarefied the MOTU reads using the rrarefy version of the vegan R package. We first rarefied to the mean number of reads of the NAT MOTUs (36,758 reads): all NIS and NAT MOTUs exceeding this threshold were rarefied to this common value. We kept unchanged the proportion of reads in each sample relative to the total reads of the MOTU total.

Before rarefaction there were 2,104 ESVs for NIS and 1,283 for NAT. With this procedure, 1,751 and 1,253 ESVs remained for NIS and NAT, respectively.

The number of ESVs/MOTU for NIS and NAT (Fig. A_2) remained significantly higher for the former (Mann-Whitney's U test, p<0.001).

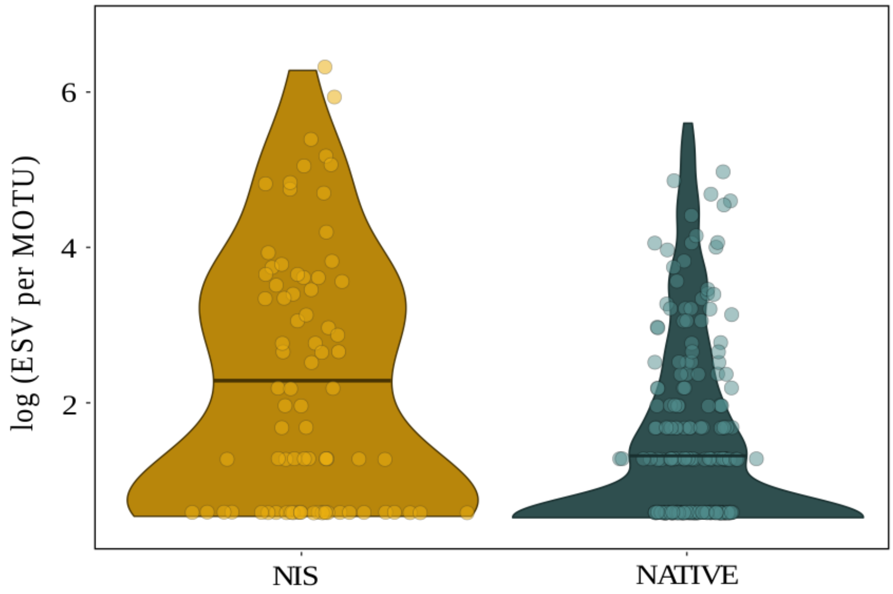

Figure A_2. Violin plots of the number of ESVs per MOTU in the NIS and NAT datasets rarefied to the mean value of the number of reads in NAT MOTUs

We performed a more stringent rarefaction to the median number of reads of the NAT dataset (354 reads). In this case only 581 and 832 ESVs remained for NIS and NAT, respectively. The results (Fig. A_3) still revealed a significantly higher number of ESVs/MOTU for NIS than for NAT MOTUs (Mann-Whitney's U test, $p<0.001$)

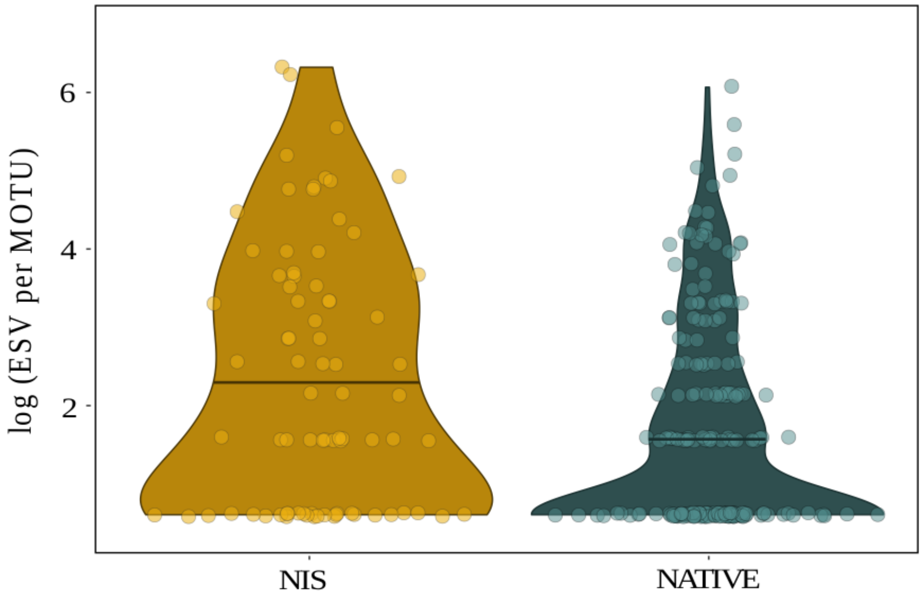

Figure A_3. Violin plots of the number of ESVs per MOTU in the NIS and NAT datasets rarefied to the median value of the number of reads in NAT MOTUs

Finally, to account for potential effects of the different sizes of the datasets (80 NIS and 235 NAT MOTUs after deletion of outliers), we performed a randomization test in which we subsampled the NAT dataset to a size of 80 and repeated the Mann-Whitney tests. We did this for the two rarefied datasets and with 100 replicates each. The results showed that 99% and 96% of the replicates of the mean- and median-rarefied datasets showed significantly higher number of ESVs per MOTU in the NIS group.

Appendix B- Comparisons between Blanes inside (BL) and Blanes outside (BO) the harbour

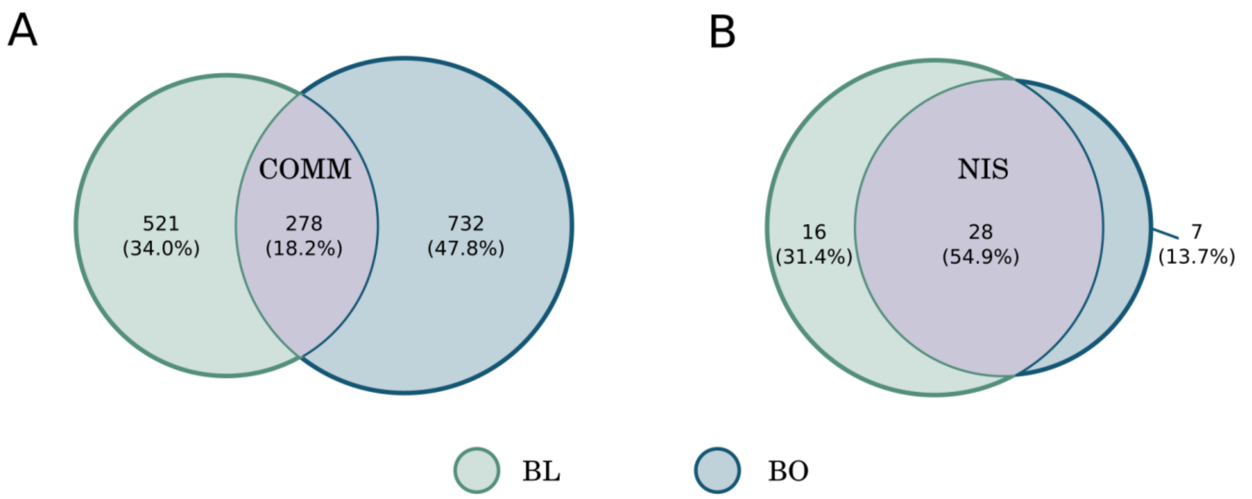

Fig. B_1. Venn diagrams showing the proportion of the number of MOTUs shared between sites for the COMM (A) and NIS (B) datasets.

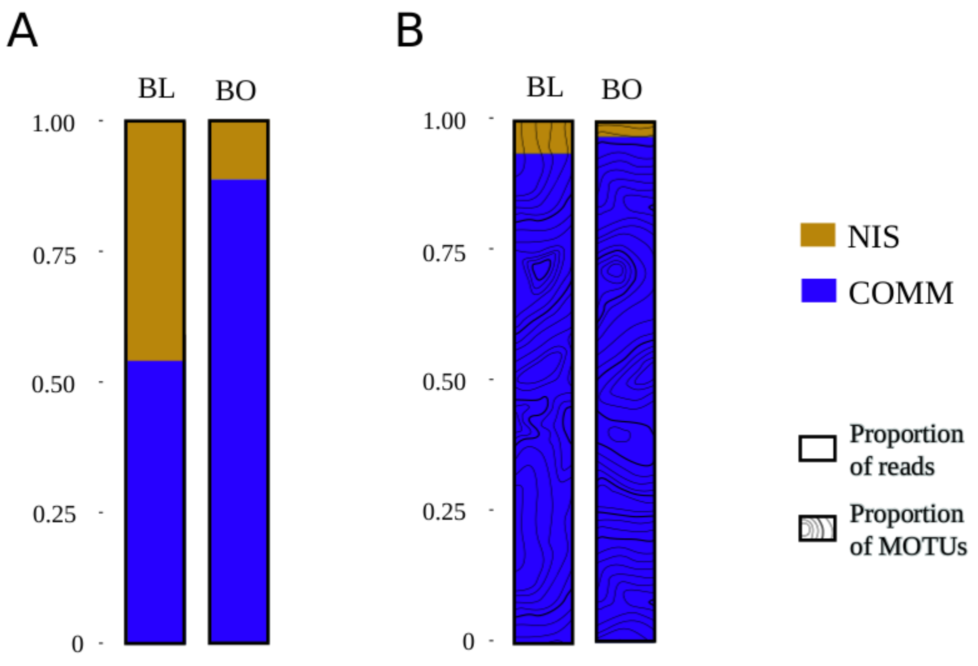

Fig. B_2. Barplots of the relative proportion of reads (A) and operational taxonomic units (MOTUs) (B) for the COMM and NIS datasets.

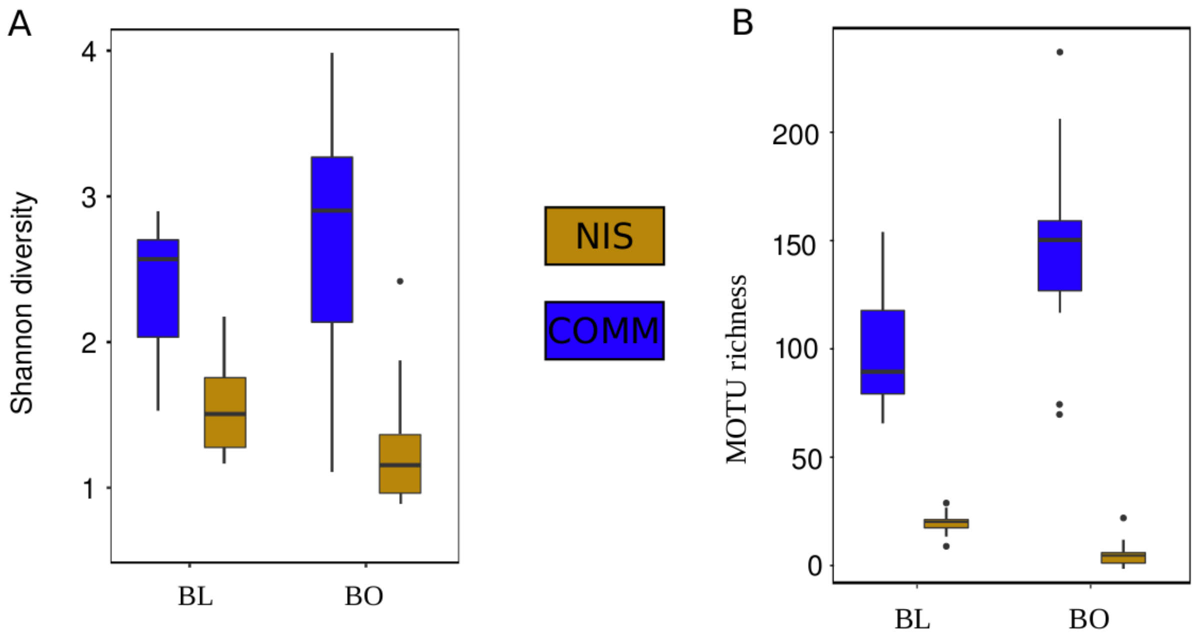

Fig. B_3. Box-plots of the values of Shannon diversity (A) and MOTU richness (B) of the COMM and NIS datasets. Horizontal lines are medians, boxes encompass the first and third quartiles, whiskers indicate 10th and 90th percentiles, and outliers are indicated as dot symbols. All comparisons were significant (Mann-Whitney tests, all $p<0.001$).

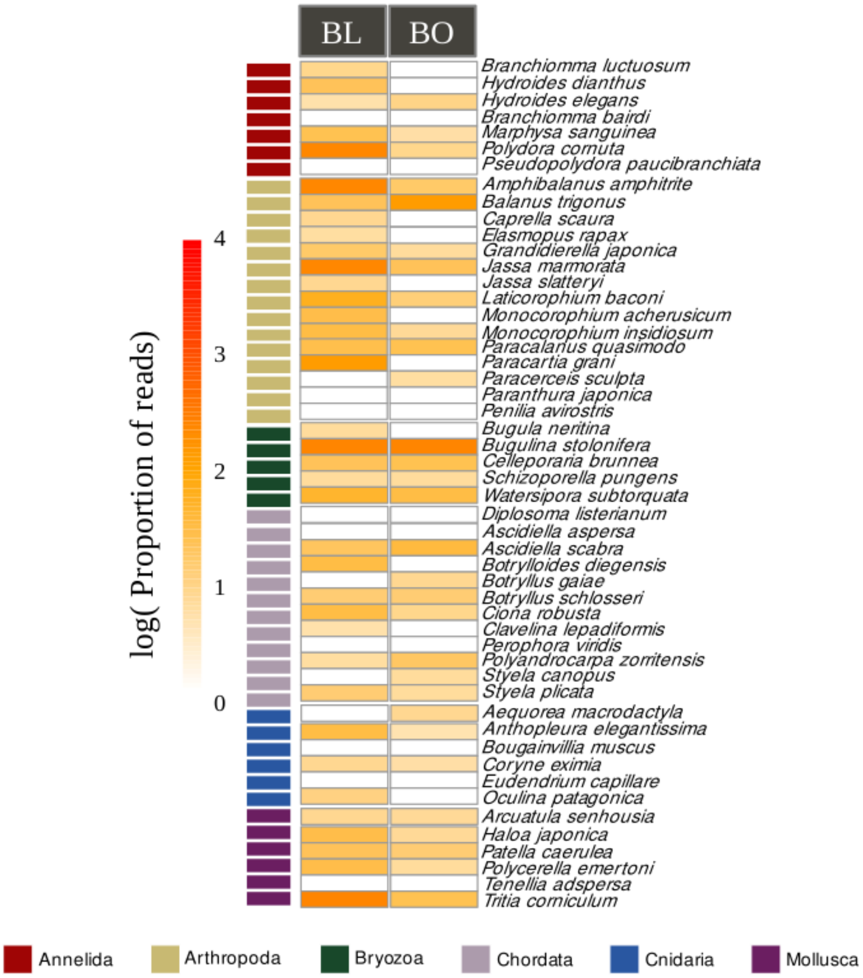

Fig. B_4- Heatmap showing relative read abundances (log-transformed) of the identified NIS in the Blanes area. Note that we have pooled molecular operational taxonomic units (MOTUs) assigned to the same nominal species. The phyla of the NIS are coded by colour. Blank cells mean absence of the species.

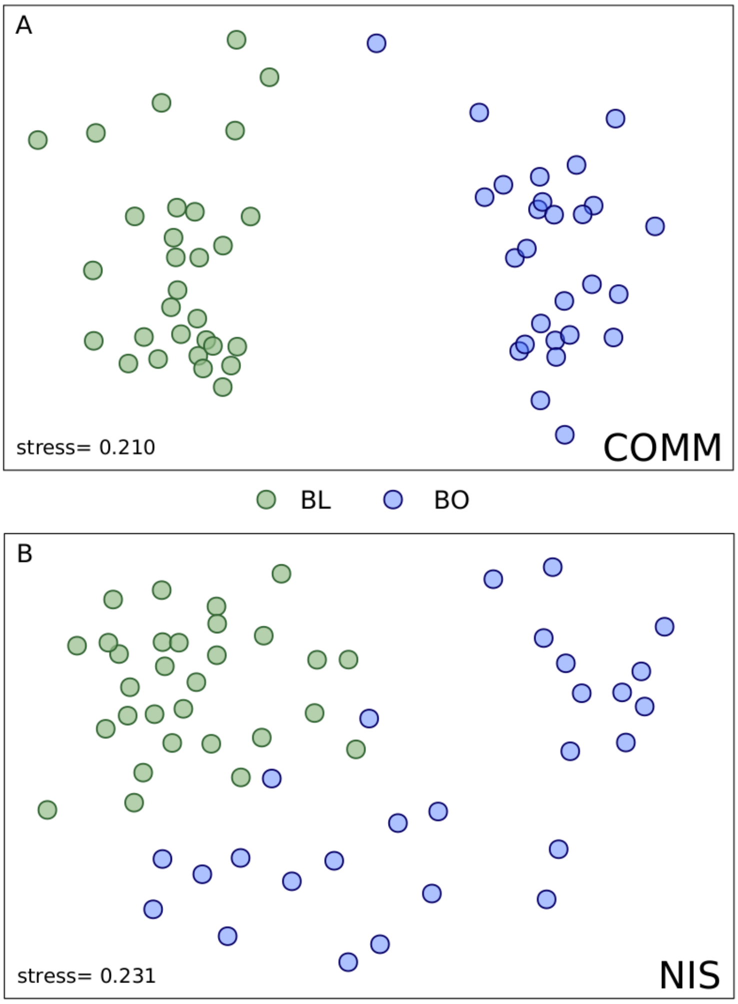

Fig. B_5 NMDS configurations for COMM (A) and NIS (B) datasets. Sites are indicated in different colours. Stresses of the final configurations are indicated.

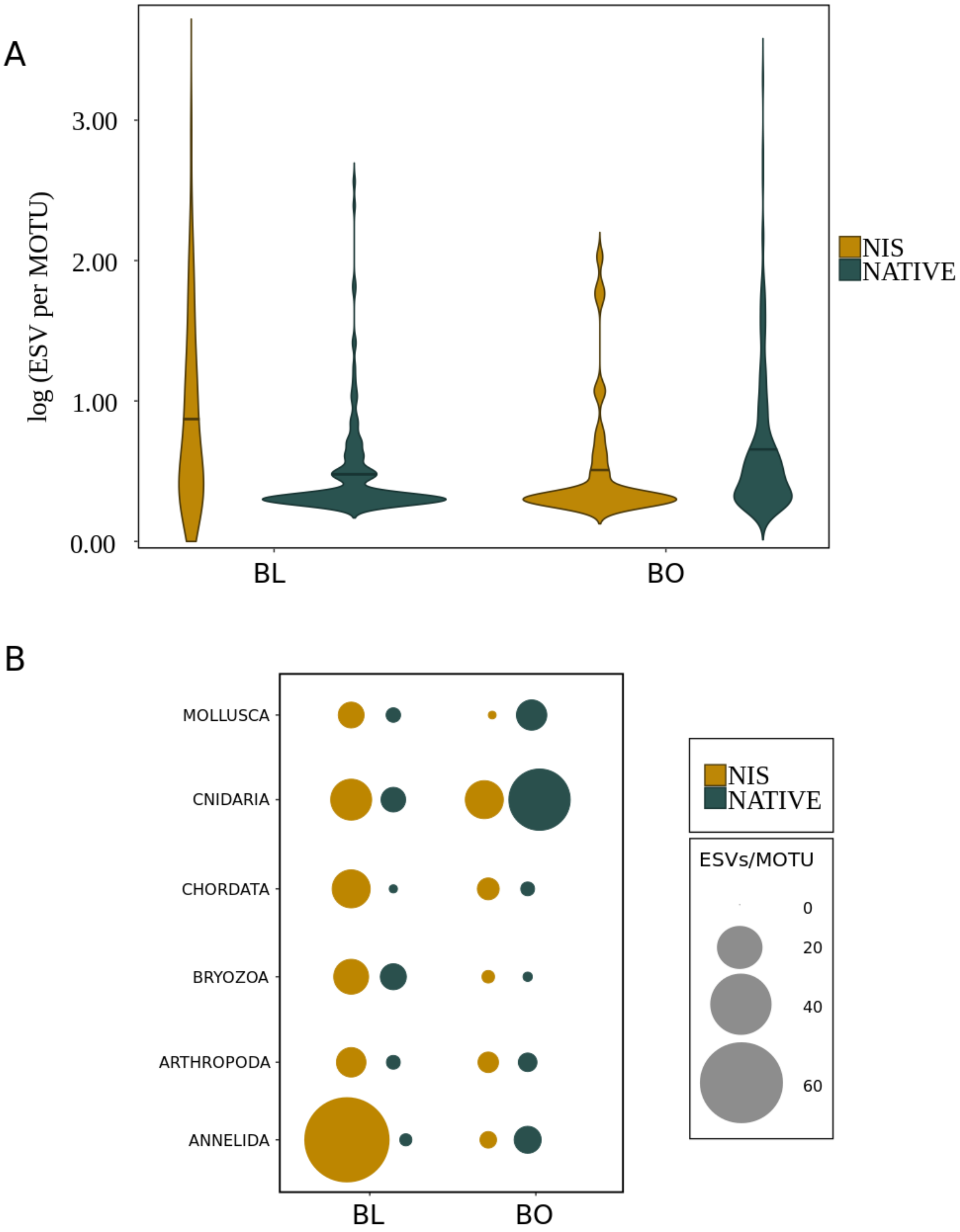

Fig. B_6 (A) Violin plots of the mean proportion of ESV per MOTU for NIS and native MOTUs. Differences were significant for BL but not for BO (Mann_Whitney tests, $p<0.001$ and $p=0.322$, respectively). (B) Bubble plots as per phyla at each site. Note natural logarithms in y axes of (A).

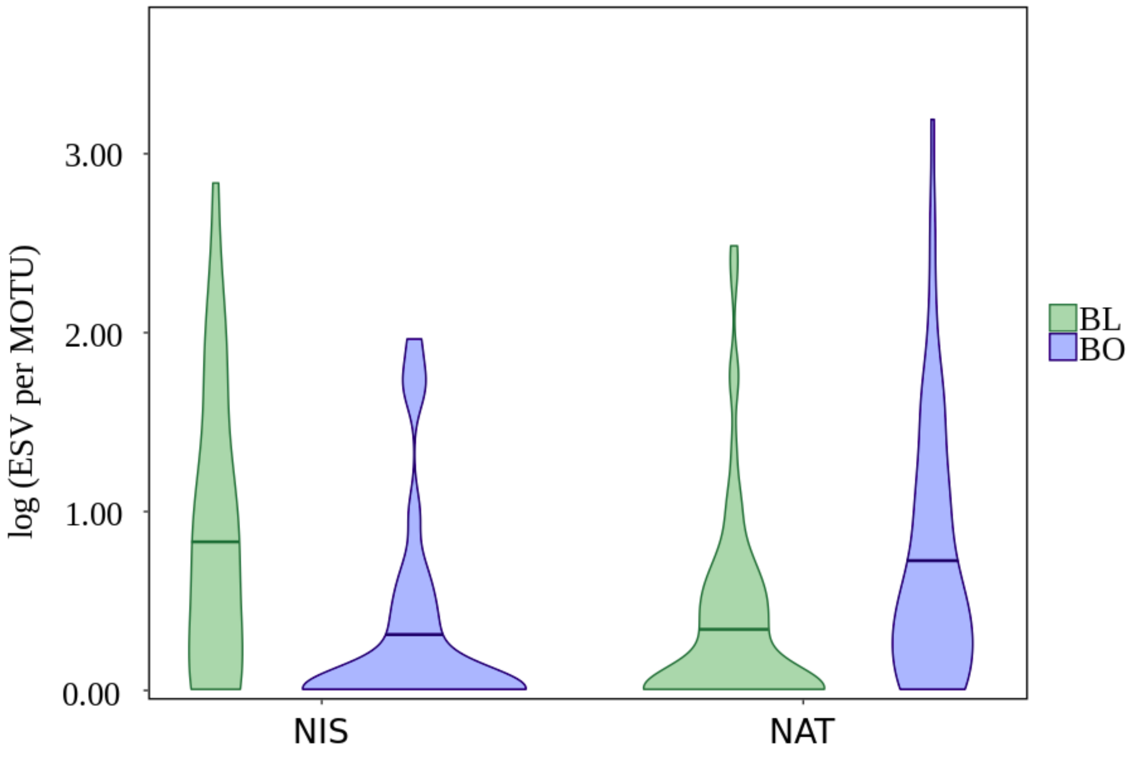

Fig. B_7. Violin plots of the mean proportion of ESV per MOTU for NIS and native MOTUs found both inside and outside the port of Blanes (31 NIS and 73 NAT MOTUs). The differences between the two locations are significant for both datasets (Wilcoxon signed-rank test, p<0.001).
